# OCTOPUS regulates BIN2 to control leaf curvature in Chinese cabbage

**DOI:** 10.1073/pnas.2208978119

**Published:** 2022-08-15

**Authors:** Xiaomeng Zhang, Wei Ma, Mengyang Liu, Xing Li, Jingrui Li, Yin Lu, Guanghuan Li, Shu Zhang, Daling Feng, Yanhua Wang, Hao Liang, Shuangxia Luo, Na Li, Aixia Gu, Shuxin Xuan, Xueping Chen, Shuxing Shen, Jianjun Zhao

**Affiliations:** ^a^State Key Laboratory of North China Crop Improvement and Regulation, Key Laboratory of Vegetable Germplasm Innovation and Utilization of Hebei, Collaborative Innovation Center of Vegetable Industry in Hebei, College of Horticulture, Hebei Agricultural University, 071000 Baoding, China

**Keywords:** leafy head, Chinese cabbage, BrOPS, brassinosteroid, leaf axial polarity

## Abstract

Heading is one of the most important agronomic traits for Chinese cabbage crops. During the heading stage, leaf axial growth is an essential process. In the past, most genes predicted to be involved in the heading process have been based on leaf development studies in *Arabidopsis*. No genes that control leaf axial growth have been mapped and cloned via forward genetics in Chinese cabbage. In this study, we characterize the inward curling mutant *ic1* in *Brassica rapa* ssp. *pekinensis* and identify a mutation in the *OCTOPUS* (*BrOPS*) gene by map-based cloning. OPS is involved in phloem differentiation in *Arabidopsis*, a functionalization of regulating leaf curvature that is differentiated in Chinese cabbage. In the presence of brassinosteroid (BR) at the early heading stage in *ic1*, the mutation of BrOPS fails to sequester brassinosteroid insensitive 2 (BrBIN2) from the nucleus, allowing BrBIN2 to phosphorylate and inactivate BrBES1, which in turn relieves the repression of *BrAS1* and results in leaf inward curving. Taken together, the results of our findings indicate that BrOPS positively regulates BR signaling by antagonizing BrBIN2 to promote leaf epinastic growth at the early heading stage in Chinese cabbage.

## Significance

Heading is a pivotal agronomic trait for Chinese cabbage, cabbage, and lettuce. The heading leaves serve as nutrient storage organs, which contribute to good quality and the economic value of leafy heads. However, the genetic basis underlying the head formation remains largely unexplored. Here, we constructed an F_2_ population with the segregation in the heading phenotype to identify the *BrOPS* gene that controls leaf curvature in a brassinosteroid-dependent manner. BrOPS interacts with BrBIN2 to modulate the phosphorylation of BrBES1 that negatively regulates the expression of leaf polarity transcription factor *BrAS1*, thereby influencing leaf curvature and heading shape in Chinese cabbage. Our data provide novel insights into leaf development and add values to future breeding of different heading types of vegetables.

The genus *Brassica* includes some of the most important vegetables worldwide. Statistically, China is the largest *Brassica* producer in the world, with 1 million ha of harvested area and 34.04 million tons of production in 2017(www.fao.org/faostat/zh). Chinese cabbage (*Brassica rapa* L. ssp. *Pekinensis* [*B. rapa*]) is a *Brassica* crop with rich leafy heads, one of the most important agronomic traits. The leafy head shape is closely associated with consumer choice and therefore directly influences the quality and economic value of Chinese cabbage crops.

Leafy head formation in Chinese cabbage depends on the curling, crinkling, and folding of leaves, leading to different overall shape. Leaf development begins at the shoot apical meristem (1) and is patterned by growth along the proximodistal, mediolateral, and abaxial-adaxial axes (2). The mechanisms governing leaf form are well studied in *Arabidopsis*. For example, more than 40 genes have been identified in the determination of abaxial-adaxial polarity (1, 3). Knowledge of leaf development in *Arabidopsis* provides a valuable foundation for predicting important regulators of leafy head formation in *Brassica* crops such as Chinese cabbage.

Heading is one of the most important agronomic traits for Chinese cabbage crops, attracting increasing attention. Reverse genetics studies suggest that the *BcpLH* (*B. rapa* ssp. *pekinensis LEAFY HEAD*) gene, a homolog of *HYPONASTIC LEAVES 1* from *Arabidopsis*, controls the initiation of leaf folding by promoting the processing of a specific subset of microRNAs (miRNAs) that coordinate leaf curvature in Chinese cabbage (4, 5). Subsequently, two auxin biosynthesis genes from an *Agrobacterium rhizogenes* plasmid pRiA4 TR region (6) were transformed into Chinese cabbage, resulting in an early folding of leaves in transgenic plants (7). In addition, several microRNAs (e.g., miRNA319, miRNA156) modulate the shape or heading time of the leafy head by posttranscriptional regulation of target genes such as *TCP4* and *SPL9* (8, 9). The reference genome of *B. rapa* ssp. *pekinensis* var. Chiffu-401-42 (http://brassicadb.cn) facilitates the study of leaf development in *B. rapa* (10). Genome resequencing of 199 *B. rapa* and 119 *B. oleracea* lines genotypes has revealed that leaf-heading morphotypes have specific polymorphic sites in genes related to the regulatory roles of four phytohormones (cytokinin, auxin, gibberellins, and jasmonic acid) and adaxial-abaxial patterning (11). Furthermore, gene transcript analysis revealed that the auxin and abscisic acid signaling genes *BrGH3.12* (*gretchen hagen3*) and *BrABF1* (*abscisic acid–responsive element binding factor1*) (12), and the auxin transport genes *BrLAX* (*like aux1*), *BrPIN* (*pin-formed*), and *BrPGP* (*ATP binding cassette subfamily B*) (13) are involved in leafy head formation. Later, using genetic and genomic approaches, quantitative trait loci (QTLs) controlling leaf development (leafy head, leaf shape, and leaf size) were identified through recombinant inbred lines and a segregating doubled haploid population (14, 15). However, to date, no genes controlling leafy head formation have been identified using a forward genetic approach of mutagenesis followed by map-based cloning in Chinese cabbage.

In a previous study, we performed ethyl methanesulfonate (EMS) to induce mutagenesis of the doubled haploid line, A03, which has an outward-curved heading pattern (16). Here, we describe one mutant from the EMS mutant with an inward curling heading pattern. We identified the mutated gene that controls the shape of the leafy head using trait segregation analysis by combining MutMap and Kompetitive Allele Specific PCR (KASP) analysis. Our analyses indicate that the *B. rapa* ssp. *pekinensis OCTOPUS* (*BrOPS*) is a key gene controlling leafy head shape. Furthermore, we describe a molecular mechanism by which BrOPS interacts with brassinosteroid insensitive 2 (BrBIN2) and recruits it to the plasma membrane, thereby inhibiting BrBIN2 phosphorylation activity on BRINSENSITIVE1 (BRI1)-EMS-SUPPRESSOR1 (BrBES1) in the nucleus, and BrBES1 transcriptionally represses the axial polarity gene *BrAS1*, which controls leaf axial growth in Chinese cabbage.

## Results

### Morphological Characteristics of *ic1* and Inheritance of the Mutant Trait.

**Fig. 1.**
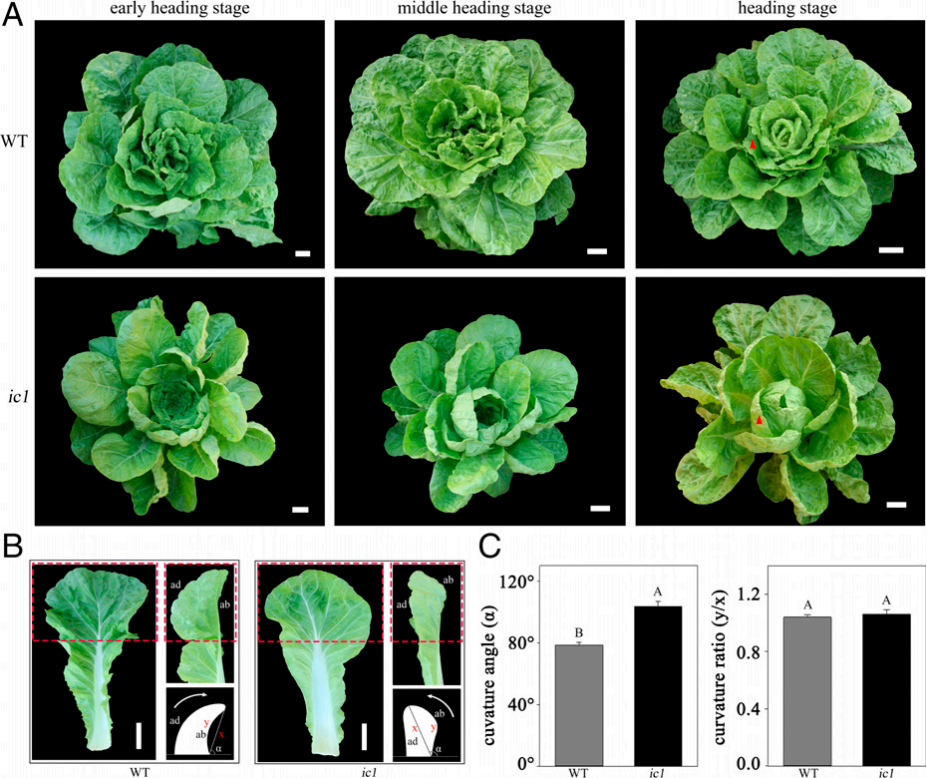
WT (A03) and *ic1* plant phenotypes. (*A*) WT and *ic1* plant phenotypes across three developmental stages (early heading, middle heading, and heading). Bar, 10 cm. (*B*) The first layer of heading leaf marked with the red triangle in (*A*). White arrows show the leaf curling direction. x and y represent the straight and curve distance from the leaf top to the petiole, respectively. α represents the curvature angle of the leaf top. Bar, 12 cm. (*C*) The curvature angle and curvature ratio of the first heading leaves in (*B*). Error bars, SD (*n* = 10). Significance was determined by ANOVA. ab, abaxial side; ad, adaxial side.

A mutant (*ic1*) with an inward curling leafy head top was isolated from an EMS mutant screen of Chinese cabbage (16). Compared with wild-type (WT) leaves, the *ic1* leaf surface was smoother, with fewer wrinkles at all five growth stages (seedling, rosette, early heading, middle heading, and heading) (Fig. 1 *A* and *B* and *SI Appendix*, Fig. S1). Beginning at the early heading stage, WT leaves showed obvious outward curvature. By contrast, the top edges of *ic1* leaves showed obvious inward curvature and developed a completely inwardly curled shape without an overlapping leafy head. At the heading stage, the curvature angle of the first heading leaf reached ∼80° in WT, while that of *ic1* developed an angle of ∼103°, but there was no difference in the curvature ratio of the heading leaf between WT and *ic1* (Fig. 1*C*). The interior leaves inside the leafy heads of *ic1* plants were looser, leading to a less compact head than that of WT (Fig. 1*A*).

Genetic analysis revealed that the phenotypic traits of all F_1_ plants were the same as those of WT. In the F_2_ population, growing in 2017 and 2018, the proportion of plants with WT and mutant traits were 3.07:1 and 3.11:1, respectively, conforming to the 3:1 ratio (χ^2^ test: χ^2^ = 0.05 and 0.08) (Table 1). These segregation ratios of F_1_ and F_2_ between WT and *ic1* plants revealed that the inward curling leaf trait was controlled by a recessive allele at a single locus.

**Table 1.** Segregation ratios of F_1_ and F_2_ between WT and *ic1* plants

Generation	Total plants	WT plants (outward-curved head)	Mutant plants (inward-curved head)	Segregation ratio	χ^2^ value
F_1_ (2017)	10	10	0	10:0	–
F_1_ (2018)	10	10	0	10:0	–
F_2_ (2017)	110	83	27	3.07:1	0.05
F_2_ (2018)	276	209	67	3.11:1	0.08

### Mutant Gene Identification.

**Fig. 2.**
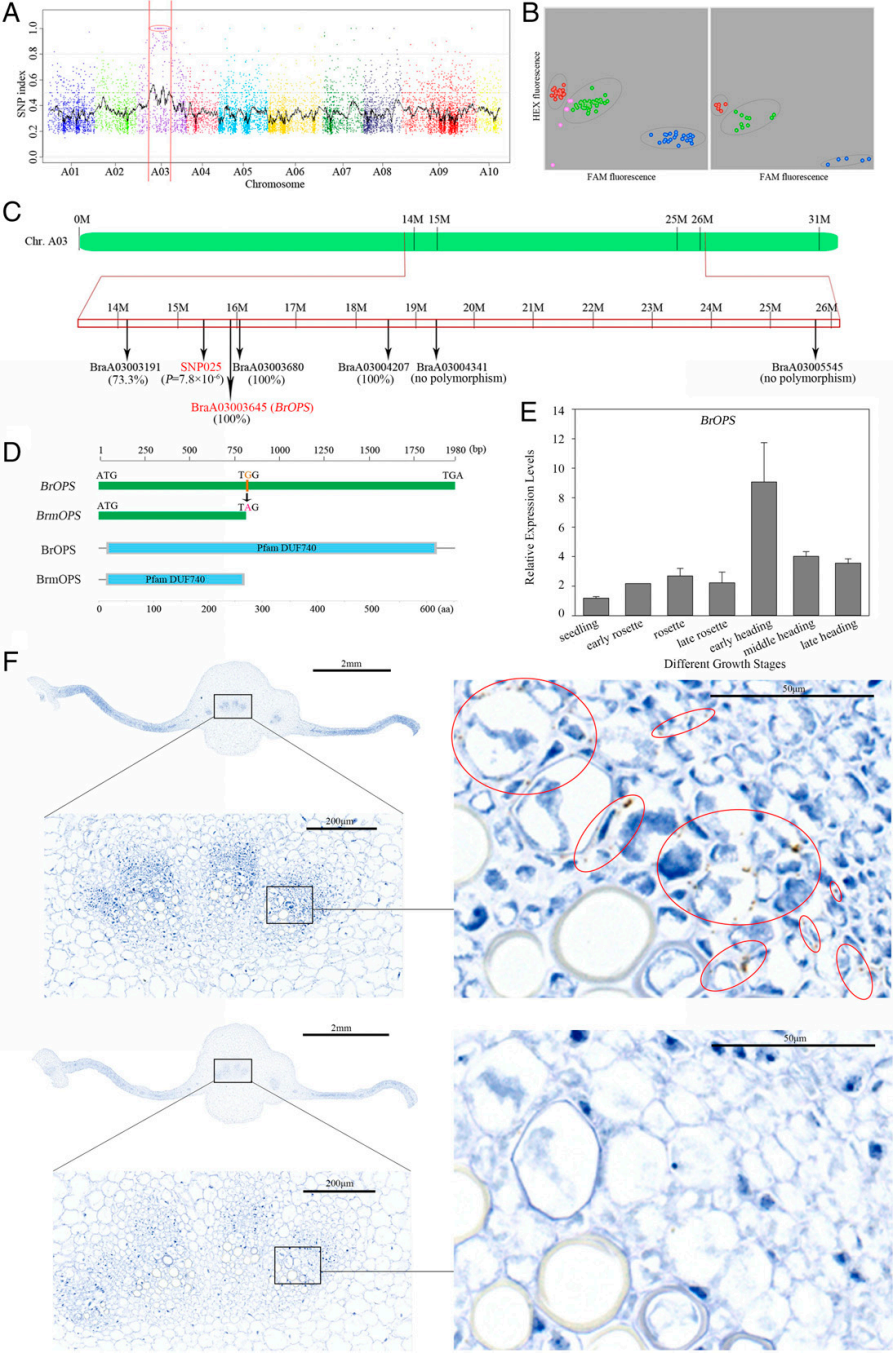
Identification of candidate loci for *ic1*. (*A*) Identification of genomic regions harboring causal mutations for *ic1* using MutMap. SNP index plots for *ic1* showing 10 chromosomes. Each symbol corresponds to an SNP, and the x-axis corresponds to the chromosomal position. The black regression line is the average value of the SNP index based on a sliding window analysis. SNPs on chromosome A03 marked by a red circle between two red lines are the predicted causal mutations for *ic1*. (*B*) Kompetitive Allele Specific PCR (KASP) genotyping assay using SNP markers in the mutant gene loci identified from MutMap in an F_2_ (WT × *ic1*) population of 96 individuals (left) and in 5 F_2_ (WT × *ic1*) individuals, 2 WT plants, 2 *ic1* lines, and 2 F_1_ lines (right). Red, mutant (*ic1*); green, heterozygous; blue, WT. pink, unknown. (*C*) The chromosomal positions of six mutant genes identified via MutMap. The key gene BraA03003645 (*BrOPS*) is labeled in red. SNP (SNP025), highlighted in red, was identified as closely associated with leafy head type in the results of target SNP and SSR-seq. The correlation values with leafy head type are indicated in parentheses beneath the SNP or gene. (*D*) Schematic diagram of the WT (*BrOPS*) and mutant (*BrmOPS*) *OPS* genes and their encoded proteins (BrOPS and BrmOPS). (*E*) The expression pattern of *BrOPS* at seven different growth stages. Error bars, SD (*n* = 3). (*F*) The tissue locations of *BrOPS* detected via RNAscope ISH at the early heading stage. The brown dots inside the red circles indicate *BrOPS*. The bottom is *BrOPS* expression detected using the sense probe of *BrOPS* as a negative control. FAM, 6-carboxy-fluorescein; HEX, hexachlorofluorescein.

To identify the candidate gene, 2 DNA sample pools from 32 F_2_ (WT × *ic1*) progeny with an inward-curved phenotype and 30 WT individuals were resequenced, and the WT genome was used as a reference genomic sequence. After single-nucleotide polymorphisms (SNPs)–index filtration (SNP index ≥0.8) of MutMap and PCR sequencing, a genomic position (16.13 to 29.34 Mb) was identified on chromosome A03 in an F_2_ population from the cross of WT and *ic1*, which included 5 nonsynonymous SNPs and 1 stop-gain substitution (Fig. 2*A* and *SI Appendix*, Fig. S2 *A–G* and Table 2).

**Table 2.** Mutation spectrum of six mutant genes selected in the *ic1* candidate genomic region identified via MutMap and PCR sequencing

Gene ID	Mutation	SNP	Mutation position Left: WT Right: mutant			WT read	Mutant-pool read
BraA03003191	Missense	16135861	Exon3	G463A	D155N	31|0	0|15
BraA03003645	Nonsense	18161644	Exon1	G809A	W270X	24|0	1|25
BraA03003680	Missense	18333685	Exon1	G661A	G221S	23|0	0|38
BraA03004207	Missense	21061306	Exon3	C1460T	A487V	33|0	2|27
BraA03004341	Missense	21964984	Exon5	G803A	S268N	27|0	0|23
BraA03005545	Missense	29344471	Exon1	G1201T	G401W	23|0	0|28

These six SNPs were used as markers in the KASP genotyping assay from the same F_2_ population. The SNP marker in BraA03003191 had a low correlation (73.3%) to the heading shape and was not considered to be a candidate gene, while SNP markers in BraA03004341 and BraA03005545 showed no polymorphism. The SNP markers in BraA03003645, BraA03003680, and BraA03004207 cosegregated with heading shape (Fig. 2*B*).

To further confirm the mapping result, an inbred line 85-1 (overlapping leaves on the head) was crossed with *ic1* to create additional F_1_ and F_2_ populations (*SI Appendix*, Fig. S3*A*). Among the target 89 accurate SNPs and 58 perfect simple sequence repeats (SSRs), 41 SNPs and 28 SSRs were identified as polymorphic (*SI Appendix*, Fig. S3*B*), and were used to identify candidate genes associated with heading type in the F_2_ population (85-1 × *ic1*). The results showed that BrSNP025 was most closely associated with the heading type (*P* = 7.8 × 10^−6^) and nearest to the SNP in BraA03003645 (Fig. 2*C*).

We further examined the candidate gene expression levels using quantitative real-time PCR at different growth stages combined with RNA sequencing (RNA-seq) using the same cDNA library on leaf sections at the early heading stage. The transcripts of BraA03003680 and BraA03004207 were not detected by quantitative real-time PCR and RNA-seq (fragments per kilobase of transcript per million mapped reads [FPKM] = 0). In addition, BraA03004341 and BraA03005545 were not detected by quantitative real-time PCR and had very low expression levels from RNA-seq (FPKM <1) at the early heading stage. In contrast, BraA03003645 was preferentially expressed at the early heading stage, which was confirmed by quantitative real-time PCR (Fig. 2*E*), consistent with a role as the key gene responsible for the leaf curling trait in Chinese cabbage heads. To confirm that BraA03003645 is the key gene responsible for the incurved head phenotype, we investigated two inward-curling inbred lines (17Q398 and 17Q430) and two outward-curling inbred lines (17Q373 and 17Q402) (*SI Appendix*, Fig. S4 *A* and *B*). One SNP, 2 insertions, and 1 deletion were identified in BraA03003645 of 17Q398, and 2 SNPs, one insertion, and 1 deletion were observed in BraA03003645 of 17Q430 (*SI Appendix*, Fig. S4*C*), but no polymorphisms were detected for BraA03003645 in 17Q373 and 17Q402, strongly suggesting that BraA03003645 plays an essential role in regulating leaf head morphology in Chinese cabbage. To confirm the tissue localization of BraA03003645 expression at the early heading stage, we performed in situ hybridization (ISH) using RNAscope ISH. BraA03003645 was expressed mainly in the meristem between the xylem and phloem, in the xylem parenchyma cells, and in undifferentiated vessel cells (Fig. 2*F* and *SI Appendix*, Fig. S5).

BraA03003645 is a homolog of *Arabidopsis OCTOPUS* (*AtOPS*) (*SI Appendix*, Fig. S6), which was called *BrOPS* for WT and *BrmOPS* for *ic1*. The coding sequence of *BrOPS* is 1980 bp without any introns and is capable of encoding a protein of 659 amino acids (Fig. 2*D*). The SNP in the *ic1* allele of BraA03003645 (*BrOPS*; G809A) is a nonsense mutation (W207X) resulting in early translation termination of BrOPS (Table 2), with part of the DUF740 domain (a domain of unknown function) (17) being lost (Fig. 2*D*). Although the whole-genome triplication (WGT) event since the separation of *B. rapa* from *Arabidopsis* yielded a second paralogous gene on chromosome A01 (BraA01004411; *BrOPS1*), the transcript level at the seedling and early heading stages of *BrOPS1* was very low and showed no difference between WT and *ic1* plants (*SI Appendix*, Fig. S7). In addition, *BrOPS1* showed no polymorphisms in either the inward-curling inbred lines (17Q398 and 17Q430) or the outward-curling inbred lines (17Q373 and 17Q402). Therefore, the phenotype of *ic1* mutant was not caused by loss-of-function of *BrOPS1*. To verify the function of *BrOPS*, stable transgenic Col-0 *Arabidopsis* plants overexpressing *BrOPS* gene exhibited the outward-curling leaves (*SI Appendix*, Fig. S8), which further confirmed the function of *BrOPS* in regulating leaf curvature. Green fluorescent protein (GFP)-BrOPS showed the polar plasma membrane–associated localization in the Col-0 transgenic lines (*SI Appendix*, Fig. S8), which is similar to the localization of AtOPS (18). Taken together, these results suggest that *BrOPS* makes contributions to the leaf curvature trait in Chinese cabbage.

In *Arabidopsis* seedlings, *OPS* loss-of-function mutants have altered vascular patterning in cotyledons and intermittent phloem differentiation in the root (18, 19), but differences in leaf curvature have not been reported. Here, we found that the mutation of *BrOPS* appeared to have no effect on phloem differentiation at the seedling and early heading stages in Chinese cabbage (*SI Appendix*, Figs. S9 and S10). It is interesting to speculate whether divergence following the WGT has enabled the acquisition of new *OPS* functions in Chinese cabbage, such as the regulation of leaf curvature.

### BrOPS Interacts with BrBIN2.

**Fig. 3.**
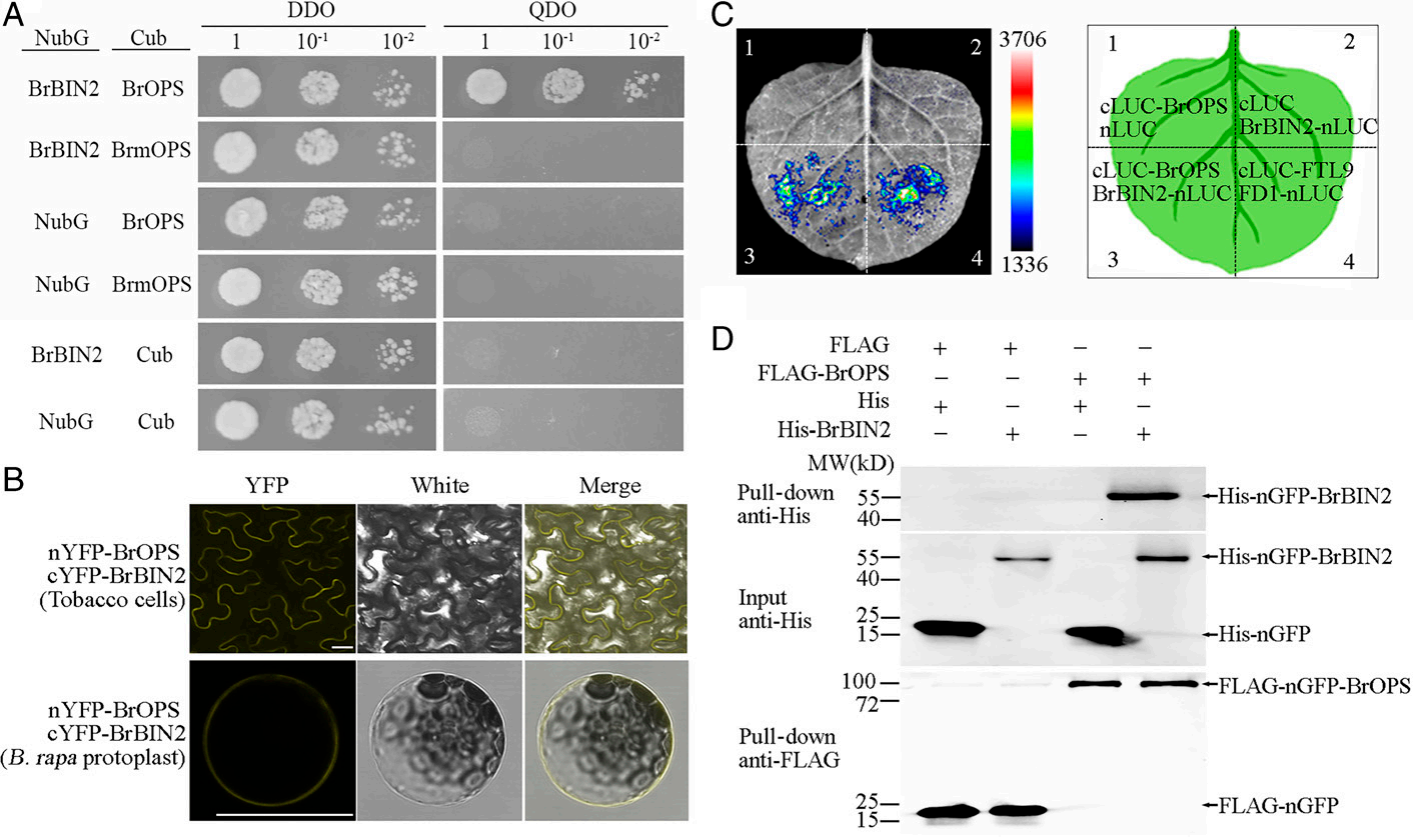
BrOPS interacts directly with BrBIN2. (*A*) BrOPS interacts with BrBIN2 in the split-ubiquitin membrane yeast two-hybrid system (quadruple dropout [QDO] + 150 ng/mL AbA medium). (*B*) BrOPS and BrBIN2 interact at the plasma membrane in plant cells. The interaction was visualized in a split-YFP assay (nYFP-BrOPS/BrmOPS and cYFP-BrBIN2) by confocal microscopy of transiently transformed *N. benthamiana* epidermal cells and Chinese cabbage protoplasts. Yellow, YFP signal. Bar, 25 μm. (*C*) BrOPS and BrBIN2 interact at *N. benthamiana* epidermal cells in a split-luciferase complementation assay. The interaction of FTL9 and FD1 is the positive control. (*D*) BrOPS interacts with BrBIN2 in a pull-down assay. DDO, double dropout.

To further analyze the function of BrOPS, its directly interacting proteins were screened from a NubG-fused cDNA library of Chinese cabbage using a split-ubiquitin membrane yeast two-hybrid (Y2H) assay. A total of 18 positive clones were detected, including 9 proteins (*SI Appendix*, Table S6). *B. rapa* BrBIN2 a known negative regulator in BR signaling (20, 21), was identified as a BrOPS interactor with the highest interaction frequency (Fig. 3*A*). The interaction between BrOPS and BrBIN2 was confirmed by bimolecular fluorescence complementation (BiFC), split-luciferase complementation, and pull-down assays (Fig. 3 *B–D*). A strong fluorescence signal in *Nicotiana benthamiana* epidermal cells and Chinese cabbage protoplast transiently coexpressing BrOPS fused to the N-terminal half of yellow fluorescent protein (nYFP-BrOPS) and BrBIN2 fused to the C-terminal half of YFP (cYFP-BrBIN2) revealed an BrOPS-BrBIN2 interaction at the plasma membrane (Fig. 3*B* and *SI Appendix*, Fig. S11*A* and *B*). However, the C-terminal truncation of BrmOPS prevented this interaction in Y2H and BiFC assays (Fig. 3*A* and *SI Appendix*, Fig. S11*A*). Meanwhile, BrOPS1 also has no direct interaction with BrBIN2 in Y2H and BiFC assays (*SI Appendix*, Fig. S12). These data demonstrate that the C-terminal of BrOPS plays an important role in the interaction with BrBIN2.

### BrOPS Recruits BrBIN2 to the Plasma Membrane.

**Fig. 4.**
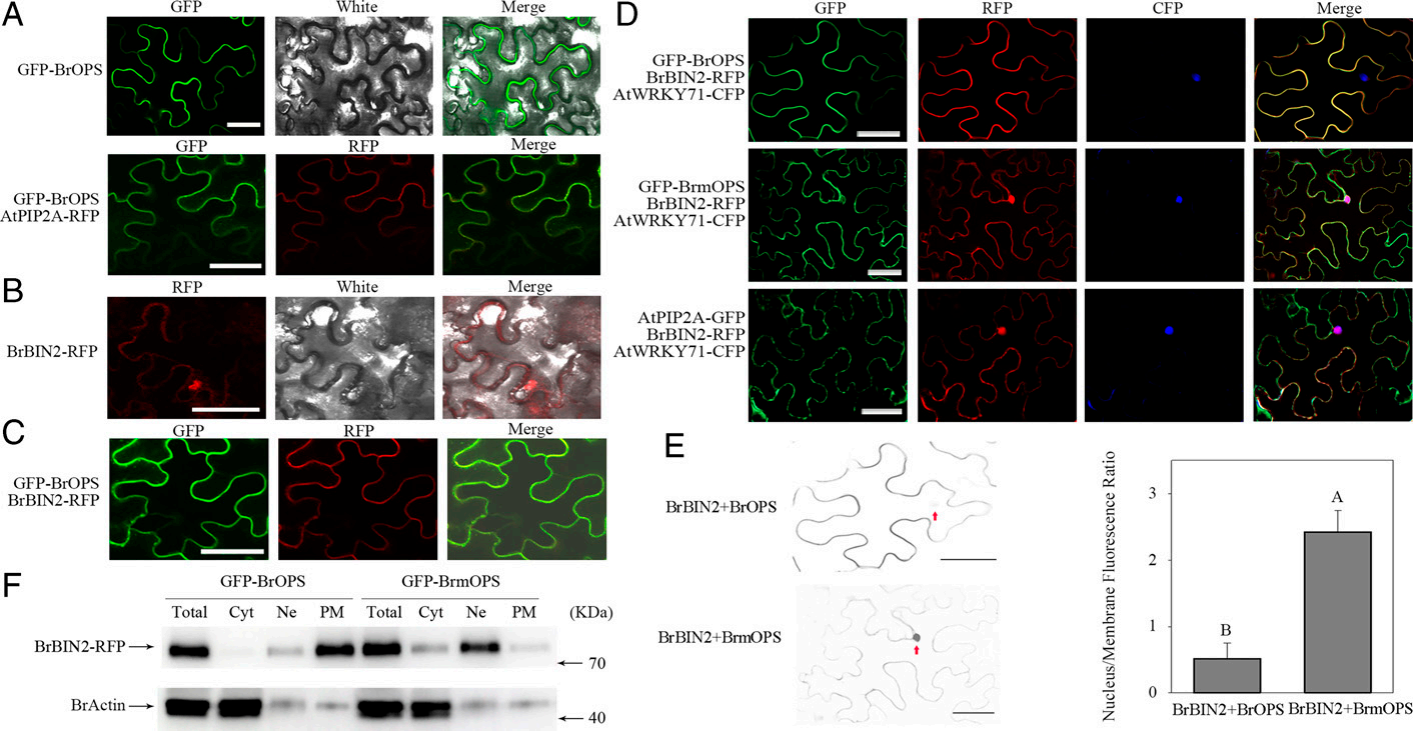
BrOPS recruits BrBIN2 to the plasma membrane. (*A*) Confocal imaging of *N. benthamiana* epidermal cells transiently GFP-BrOPS, and coexpressing GFP-BrOPS and the plasma membrane marker protein AtPIP2A-RFP (red fluorescent protein). (*B*) Confocal imaging of *N. benthamiana* epidermal cells transiently expressing BrBIN2-RFP. (*C*) Confocal imaging of *N. benthamiana* epidermal cells transiently coexpressing GFP-BrOPS and BrBIN2-RFP. (*D*) BrBIN2-RFP localization in the presence of BrOPS, BrmOPS, or AtPIP2A with AtWRKY71 (a nuclear localized protein) visualized by confocal microscopy. (*E*) The fluorescence signal in (*D*) is displayed as inverted gray values for clearer visualization. Red arrowheads show nuclear signals. Subcellular distribution changes in BrBIN2 were quantified in the presence of BrOPS. Nucleus/plasma membrane ratio of mean fluorescence intensity measured by ImageJ. Error bars, SD (*n* = 10). Significance was determined by ANOVA. (*F*) Nuclear/cytosolic/plasma membrane fractionation assay of BrBIN2 in the presence or absence of BrOPS using western blot analysis of the *N. benthamiana* transient expression system. Bar, 50 μm.

The subcellular proteins can provide insight into their functional role. When GFP-BrOPS was transformed alone into *N. benthamiana* epidermal cells and Chinese cabbage protoplasts, BrOPS was located at the plasma membrane, colocalizing with the plasma membrane marker AtPIP2A-RFP and AHA1-mCherry (22, 23) (Fig. 4*A* and *SI Appendix*, Fig. S13*A–C*). Different from the localization of BrOPS, BrmOPS displayed a strong fluorescence signal in the plasma membrane, nuclear, and cytoplasm (*SI Appendix*, Fig. S13*A–C*). However, BrBIN2 was observed primarily in the nucleus, with a weak signal at the plasma membrane (Fig. 4*B* and *SI Appendix*, Fig. S13*D*). Interestingly, transient assays coexpressing BrOPS and BrBIN2 in *N. benthamiana* epidermal cells and Chinese cabbage protoplasts showed that BrOPS and BrBIN2 colocalized in the plasma membrane (Fig. 4*C* and *SI Appendix*, Fig. S13*E*), which was consistent with the findings of BiFC showing their interaction in the plasma membrane (Fig. 3*B* and *SI Appendix*, Fig. S11*A* and *B*). Next, we performed a transient transformation of BrBIN2-RFP with GFP-BrOPS/GFP-BrmOPS/AtPIP2A-GFP and AtWRKY71-CFP (cyan fluorescent protein) (nuclear control) (24) in *N. benthamiana* and BrBIN2-RFP with GFP-BrOPS/GFP-BrmOPS in Chinese cabbage protoplasts, while BrBIN2-RFP alone or with GFP-BrmOPS exhibited a strong signal in the nucleus and a lower signal at the plasma membrane (Fig. 4*D* and *SI Appendix*, Fig. S13*E*). By contrast, in the presence of GFP-BrOPS, BrBIN2 localization exhibited a higher signal at the plasma membrane and a lower signal in the nucleus (Fig. 4*D* and *SI Appendix*, Fig. S13*E*). This result was confirmed by quantifying the fluorescence intensity in the nucleus and plasma membrane under the two conditions (Fig. 4*E*). Western blot analysis also showed the same effect of BrOPS on BrBIN2 localization (Fig. 4*F* and *SI Appendix*, Fig. S14). These results indicate that in the presence of GFP-BrOPS, BrBIN2-RFP is sequestered at the plasma membrane, thereby reducing its abundance in the nucleus.

### BrBIN2 Regulates Expression Patterns of *BrAS1* in a BrBES1-Dependent Manner.

**Fig. 5.**
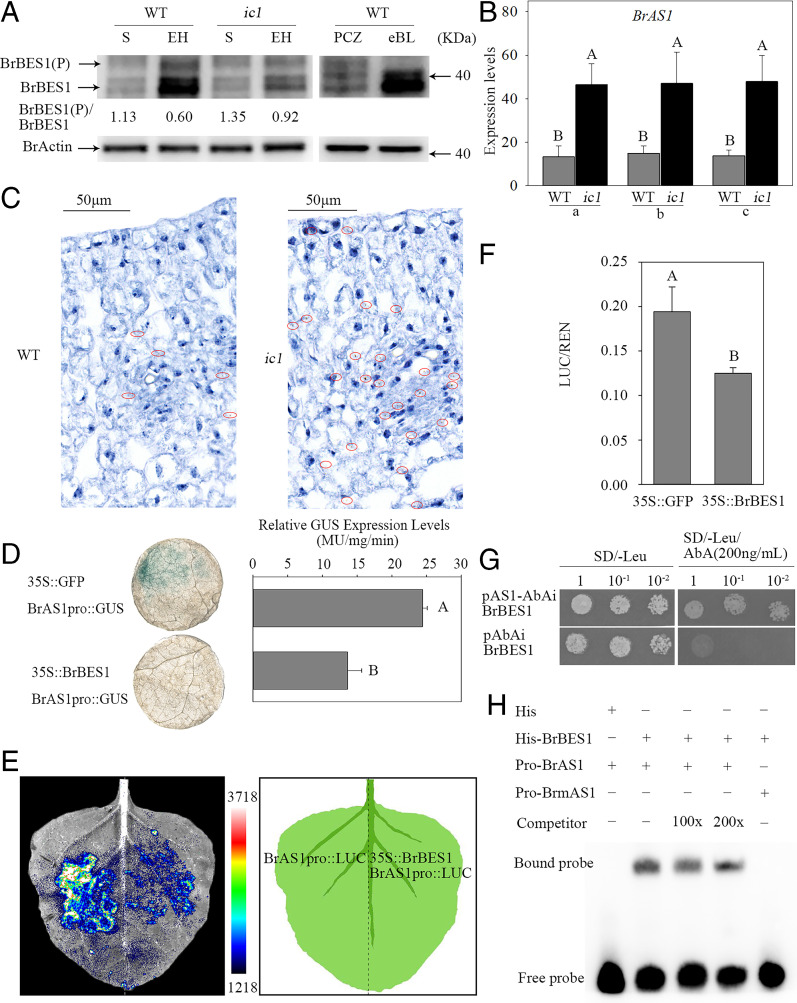
BrBIN2 regulates expression patterns of *BrAS1* in a BrBES1-dependent manner. (*A*) The phosphorylation levels of BrBES1 in WT and *ic1* plants at the seedling (S) and early heading (EH) stages. The BES1 antibody was used to detect the abundance and phosphorylation of BrBES1 in Chinese cabbage leaves. The actin antibody was used to detect the abundance of BrActin, as a control. The ratio of phosphorylated/dephosphorylated BrBES1 represented average value from three repeats. The repeat included five leaves each from independent plants. (*B*) Transcript level of *BrAS1* in different leaf sections. a, b, and c correspond to the leaf samples shown in *SI Appendix*, Fig. S7*A*. (*C*) The tissue locations of *BrAS1* detected via RNAscope ISH at the early heading stage. The brown dots inside the red circles indicate *BrAS1*. (*D*) *N. benthamiana* leaves stained for *BrAS1pro::GUS* expression upon coexpression with 35S::GFP (upper) or 35S::BrBES1 (lower) and quantification of *BrAS1pro::GUS* expression in the presence of 35S::GFP or 35S:: BrBES1, respectively. Error bars, SD (*n* = 12). Significance was determined by ANOVA. (*E*) The luminescence signal produced by BrAS1pro::LUC in the presence or absence of 35S::BrBES1 in *N. benthamiana*. (*F*) Firefly and Renilla luciferases were measured in the presence of 35S::GFP or 35S::BrBES1 using a dual-luciferase reporter system. (*G*) BrBES1 bound to the *BrAS1* promoter in a Y1H assay. (*H*) BrBES1 bound to the E-box of the *BrAS1* promoter in an EMSA.

We note from previous studies that BIN2 phosphorylates two downstream transcription factors, BES1/brassinazole-resistant 1 (BZR1) in the nucleus, to repress the BR response (25–28). According to the *BrOPS* preferential expression at the early heading stage (Fig. 2*E*), and BrOPS sequestering BrBIN2 at the plasma membrane (Figs. 3*B* and 4 *C–F*), we wonder whether BrBIN2 sequestration at the plasma membrane affects the phosphorylation status of BrBES1 and BrBZR1. The results showed that BrBZR1 phosphorylation status maintained similar levels in WT and *ic1* mutant at the seedling and early heading stages (*SI Appendix*, Fig. S15). Similarly, phosphorylated BrBES1 at the seedling stage of WT and *ic1* mutant accumulated at the same level (Fig. 5*A*). However, at the early heading stage, the abundance of phosphorylated BrBES1 in *ic1* was significantly higher than in WT (Fig. 5*A*). In addition, the concentrations of brassinolide (BL) were significantly higher at the early heading stage than at the seedling stage in both WT and *ic1* (*SI Appendix*, Fig. S16). These data indicate that high levels of BL concentration and *BrOPS* transcript promote the accumulation of unphosphorylated BrBES1 at the early heading stage.

Leaf curvature and axial polarity are essential for the formation and shape of the leafy head in Chinese cabbage (8). Therefore, we focused on differences in the expression of axial polarity genes in three leaf sections at the early heading stage in *ic1* by RNA-seq and quantitative real-time PCR (Fig. 5*B* and *SI Appendix*, Fig. S17). Six axial polarity genes, including four *KNOX* genes (*BrKNAT5*, *BrKNAT4.1*, *BrKNAT4.2*, and *BrKAN1*), *BrAS1*, and one *HD-ZIPIII* gene (*BrHB8*) (1), were differentially expressed in *ic1* leaves compared to WT leaves. In *ic1* leaves, the expression levels of the four *KNOX* genes and *BrAS1* were up-regulated, whereas that of *BrHB8* was down-regulated (Fig. 5 *B* and *C* and *SI Appendix*, Figs. S17*B* and S18). Interestingly, *cis*-acting elements (CANNTG, E-boxes) that are the binding targets of BES1 (21) were found in the promoters of the six axial polarity genes, but no binding motif of BZR1 was found.

To test whether axial polarity genes were regulated by BrBES1, promoters of three axial polarity genes (*BrKNAT4.1*, *BrKNAT4.2*, and *BrAS1*), which possess high transcript levels and obvious expression differences in three leaf sections between WT and *ic1* (*SI Appendix*, Figs. S17*B* and S19*A*), were selected and cloned. When cotransformed into *N. benthamiana*, 35S::BrBES1 down-regulated the expression of *BrAS1pro::GUS* but had no effect on the expression of the *BrKNAT4.1pro::GUS* and *BrKNAT4.2pro::GUS* (Fig. 5*D* and *SI Appendix*, Fig. S19 *B* and *C*). The expression of *BrAS1pro::LUC* was also repressed by 35S::BrBES1 in *N. benthamiana* (Fig. 5 *E* and *F*). In addition, the yeast one-hybrid (Y1H) assay suggested that BrBES1 bound directly to the *BrAS1* promoter (Fig. 5*G*), and an electrophoretic mobility shift assay (EMSA) indicated that BrBES1 bound to the E-box of the *BrAS1* promoter (Fig. 5*H*). By contrast, BrBES1 did not bind to the *BrKNAT4.1* and *BrKNAT4.2* promoters in the Y1H assay (*SI Appendix*, Fig. S19*D*). These results indicate that BrBES1 bound to the E-box motif of the *BrAS1* promoter and repressed *BrAS1* expression. The transcript level of *BrAS1* was lower in WT than in *ic1*, leading to leaf outward curling consistent with that in *Arabidopsis* (29). To further confirm the roles of *BrAS1* in leaf heading types, the transcript levels of *BrAS1* in two inward-curling inbred lines (17Q398 and 17Q430) and two outward-curling inbred lines (17Q373 and 17Q402) were assessed at the heading stage. The expression levels of *BrAS1* were much lower in outward-curling inbred lines than in inward-curling inbred lines (*SI Appendix*, Figs. S20 and S21).

Taken together, these results suggest that BrBIN2 phosphorylates and inactivates BrBES1, which in turn relieves the repression of *BrAS1* and results in leaf inward curving in *ic1*.

### *ic1* Mutant Plants Showed Reduced Sensitivity in BR.

**Fig. 6.**
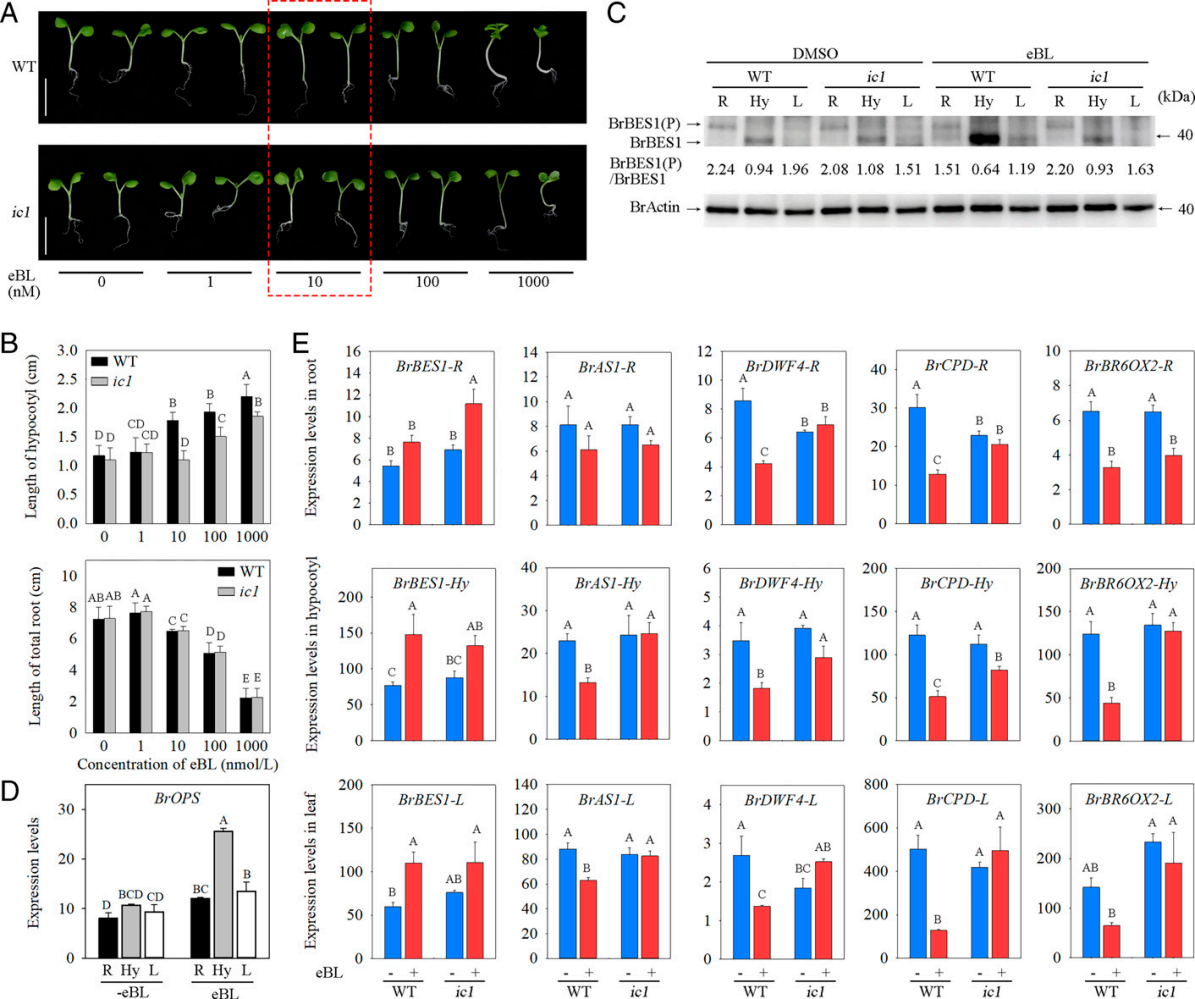
*ic1* mutant showed insensitivity to BR. (*A*) Seven-day-old in WT and *ic1* mutant seedlings grown on Murashige & Skoog (MS) medium with 1% sucrose supplemented with different concentrations of eBL ranging from 1 to 1000 nM. Bar, 2 cm. The WT and *ic1* mutant seedlings treated with 10 nM eBL in the red box were used to assess the phosphorylation status of BrBES1 and transcript levels of *BrOPS* and BR synthesis genes in roots, hypocotyls, and leaves. (*B*) Hypocotyl (*Top*) and total root (*Bottom*) length of seedlings shown in (*A*). Error bars, SD (*n* = 30). Significance was determined by ANOVA. (*C*) The phosphorylation levels of BrBES1 in roots (R), hypocotyls (Hy), and leaves (L) of WT and *ic1* seedlings treated with DMSO or eBL. The BES1 antibody was used to detect the abundance and phosphorylation of BrBES1 in Chinese cabbage roots, hypocotyls, and leaves. The actin antibody was used to detect the abundance of BrActin, as a control. The ratio of phosphorylated/dephosphorylated BrBES1 represented average value from three repeats. Each repeat included 15 roots, hypocotyls, and leaves from independent seedlings. (*D*) Transcript levels of *BrOPS* in roots, hypocotyls, and leaves of WT seedlings treated with DMSO or eBL. Error bars, SD (*n* = 3). Significance was determined by ANOVA. (*E*) Transcript levels of *BrBES1*, *BrAS1*, *BrDWF4*, *BrCPD*, and *BrBR6ox2* in roots, hypocotyls, and leaves of WT and *ic1* seedlings treated with DMSO or eBL. Error bars, SD (*n* = 3). Significance was determined by ANOVA.

Controlling the BR signaling pathway can regulate the growth of the hypocotyl (26, 27). Therefore, the hypocotyl length of WT and *ic1* mutants was examined. Without any treatment, the hypocotyl in the *ic1* mutant maintained a size similar to that of WT under 16-h light/8-h dark photoperiod or dark conditions (Fig. 6 *A* and *B* and *SI Appendix*, Fig. S22*A* and *B*). With the propiconazole (PCZ, a BR biosynthesis inhibitor) (30) treatment under dark conditions for 7 days, we found that WT plant and *ic1* mutant also displayed no difference in hypocotyl growth (*SI Appendix*, Fig. S22 *A* and *B*). This observation is likely caused by both the reduced BR content and the lower expression of the *BrOPS* at the seedling stage compared to the heading stage (Fig. 2*E* and *SI Appendix*, Fig. S16). Then, we treated the seedlings of WT and *ic1* mutant with BR (eBL) and assessed the phenotypes associated with BR treatment. We found that the *ic1* mutant was insensitive to a low concentration (10 nM) of eBL and showed lower sensitivity than WT plants to high concentrations (100 and 1,000 nM) of eBL (Fig. 6 *A* and *B*). Meanwhile, we assessed the BrBES1 abundance, the ratio of phosphorylated/dephosphorylated BrBES1 and transcript levels of BR synthesis genes in hypocotyls of *ic1* and WT treated with 10 nM eBL. The signals in western blot clearly showed that the abundance of BrBES1 was significantly decreased in *ic1* mutant hypocotyl compared to WT, while the ratio of phosphorylated/dephosphorylated BrBES1 increased in *ic1* mutant (Fig. 6*C* and *SI Appendix*, Fig. S23). In addition, the expression of *BrDWF4* and *BrBR6ox2* was feedback suppressed in WT, but not obviously suppressed in *ic1* (Fig. 6*E*). It is concluded that *BrOPS* plays an important role in BR signaling.

However, *ic1* seedlings exhibited shortened roots and outward curling cotyledons similar to WT in response to exogenous eBL (Fig. 6 *A* and *B*). To decipher the underlying mechanism of different tissues of the *ic1* mutant, including roots, hypocotyls, and leaves, in response to BR, we assessed the transcript levels of *BrOPS* and the abundance of BrBES1 in roots, hypocotyls, and leaves, respectively. As shown in Fig. 6*D*, the expression level of *BrOPS* was much higher in hypocotyls than that in roots and leaves after eBL treatment. Similarly, the abundance of BrBES1 protein was also higher in hypocotyls than that in roots and leaves (Fig. 6*C*). Given that BrOPS positively regulates the BR signaling pathway, the mutation of *BrOPS* reduces the abundance of dephosphorylated BrBES1, resulting in a higher ratio of phosphorylated/dephosphorylated BrBES1 in the hypocotyl of *ic1* that causes the different phenotypes in the hypocotyl (Fig. 6*C*). *BrOPS* transcript levels and BrBES1 abundance are significantly lower, and the ratio of phosphorylated/dephosphorylated BrBES1 is obviously higher in the roots and leaves than that in the hypocotyls, which may explain the lack of an obvious difference between *ic1* and WT plants in the roots and leaves after eBL treatment (Fig. 6 *C* and *D*).

Notably, ectopic overexpression of *BrOPS* in *bri1-116* (31), a BR-insensitive *Arabidopsis* mutant, partially suppressed the *bri1-116* phenotypes (*SI Appendix*, Fig. S24). These findings suggest that *BrOPS* was involved in BR signaling with a positive regulation.

## Discussion

Leafy heads of vegetables, such as Chinese cabbage (*B. rapa* L. ssp. *pekinensis*), cabbage (*B. oleracea* L. ssp. *capitata*), and lettuce (*Lactuca sativa* L. var. *capitata*) are composed of yellow-green, extremely crinkly, and inward curved leaves (8). Head shape and color are subjects of consumer preference, which consequently determines the quality and commercial value of these crops (8). Heading thus constitutes an important agronomic trait that has been the subject of intensive study. In *Arabidopsis*, studies have implicated miRNA regulation of *TCP* transcription factor expression in the determination of leaf shape (32). In Chinese cabbage, a natural variation (QTL) approach has identified a similar regulatory network in the control of heading (8, 14). However, few studies on gene function in the control of head shape have applied forward genetics in Chinese cabbage.

Studies on the phytohormone regulation of leafy head formation have emphasized auxin (7, 11, 13). Although BRs, plant steroid hormones, are known to affect many processes such as hypocotyl and stem elongation, leaf development, pollen tube growth, vascular differentiation, senescence, and photomorphogenesis (33–37), a role for BR signaling in the regulation of leaf heading in Chinese cabbage was not demonstrated previously. Here, 100 μM PCZ was applied twice to WT and *ic1* mutant plants in pots at the rosette stage. Thirty days after treatment, newly expanded leaves of PCZ-treated WT plants displayed inward-curling phenotype similar to *ic1* mutant (*SI Appendix*, Fig. S25). Interestingly, we observed that both types of plants tend to head as well as with phenotypes of wrinkled leaves and slow leaf growth compared to untreated plants. These results confirm the BR signaling that regulates the heading phenotype of Chinese cabbage.

In *Arabidopsis*, OPS is involved in phloem differentiation (18, 19). *OPS* loss-of-function mutants showed altered vascular networks in cotyledons and intermittent phloem differentiation in the root (18, 19), but the effect of *OPS* on leaf curvature is never reported. Here, we identified a loss-of-function mutation of *B. rapa OPS* that confers inward curvature of head leaves, resulting in a leafy head shape distinct from that of WT (Fig. 1). However, we detected no effect of the *BrOPS* mutation on phloem differentiation (*SI Appendix*, Figs. S9 and S10), indicating that BrOPS has been deployed in a new role in Chinese cabbage. As a consequence of the whole genome triplication that occurred in *B. rapa* after its separation from *Arabidopsis*, there are two paralogous copies of *OPS* in the *B. rapa* genome. This duplication would permit neofunctionalization of one copy, allowing *OPS* to adopt a different function in the regulation of leafy head shape, while we speculate the *BrOPS1* has maintained a role in the regulation of vascular differentiation.

In *Arabidopsis*, the overexpression of *OPS* activates the BR pathway, resulting in elongated hypocotyls and curly cotyledons, mimicking WT seedlings grown on medium containing 1 μM eBL (38). In our study, we found that the enhanced hypocotyl growth in WT and *ic1* mutant displayed a difference under different concentrations of eBL ranging from 1 to 1000 nM (Fig. 6). Increased hypocotyl growth was observed when WT plants were exposed to 10 nM eBL, but this phenotype was observed in *ic1* mutant exposed to a higher concentration of 100 nM eBL (Fig. 6). At 1,000 nM concentration of eBL, the hypocotyl of *ic1* mutant was shorter than WT (Fig. 6). These observations indicated that *ic1* mutant was hyposensitive to BR, and thus BrOPS plays a positive role in BR response.

In *Arabidopsis*, *OPS* activates BR signaling by sequestering the GSK3 kinase BIN2 at the plasma membrane. This relieves the inhibitory phosphorylation of BES1 in the nucleus, allowing the BES1 transcription factor to repress the expression of target genes (38). BrOPS seems to work in the same way by directly interacting with BrBIN2 and sequestering BrBIN2 to the plasma membrane (Figs. 3 and 4), resulting in the accumulation of unphosphorylated BrBES1 (Fig. 5*A*). In particular, we have shown that BrBES1 represses the expression of one axial polarity gene, *BrAS1*, although not of other axial polarity genes misexpressed in the *ic1* mutant. However, this may have been regulated by other transcription factors.

**Fig. 7.**
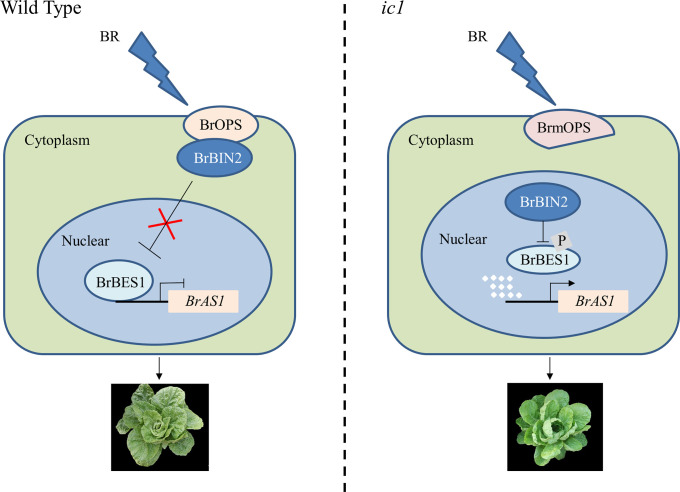
Simplified scheme of the BR pathway at the early heading stage in WT (*Left*) or *ic1* (*Right*) plants. In the presence of BR at the early heading stage, when *BrOPS* is highly expressed, BrOPS interacts directly with BrBIN2 at the plasma membrane, blocking BrBIN2 accumulation in the nucleus. Unphosphorylated BrBES1 accumulates in the nucleus, inhibiting transcription of *BrAS1*. Low expression of *BrAS1* causes leaf outward curling in the WT. In the absence of *BrOPS* in *ic1*, BrBIN2 accumulates in the nucleus, phosphorylating the transcription factor BrBES1. Phosphorylated BrBES1 releases the repression of *BrAS1* transcription, leading to leaf inward curling in *ic1*.

In conclusion, our forward genetic approach has revealed a different function for OPS in BR signaling to regulate leaf curvature and hence head shape in *B. rapa.* In the presence of BR at the early heading stage, when *BrOPS* is highly expressed, BrOPS interacts directly with BrBIN2 at the plasma membrane, blocking BrBIN2 accumulation in the nucleus. Unphosphorylated BrBES1 accumulates in the nucleus, inhibiting the transcription of *BrAS1*. Low expression of *BrAS1* causes leaf outward curling in the WT (Fig. 7). In the absence of *BrOPS* in *ic1*, BrBIN2 accumulates in the nucleus, phosphorylating the transcription factor BrBES1. Phosphorylated BrBES1 releases the repression of *BrAS1* transcription, leading to leaf inward curling in *ic1* (Fig. 7). This role had not been anticipated from work in *Arabidopsis*, emphasizing the importance of forward genetic analyses in nonmodel organisms. From a more practical perspective, our results also suggest other avenues for the manipulation of leaf head shape in Chinese cabbage and potentially in other heading vegetables.

## Materials and Methods

Details of plant materials and growth conditions are provided in the *SI Appendix* and *SI Appendix*, *Materials and Methods*. Inheritance of the mutant trait, candidate mutant genes mapped by MutMap, KASP, and target SSR and SNP-seq; BrOPS interaction with BrBIN2 by split-ubiquitin membrane Y2H assay, BiFC, split-luciferase complementation, and pull-down assays; BrOPS recruiting BrBIN2 to the plasma membrane by transient gene expression in *N. benthamiana* cells and Chinese cabbage protoplasts, and western blot; BrBIN2 regulating expression patterns of *BrAS1* in a BrBES1-dependent manner by western blot, RNA-seq, quantitative real-time PCR, RNAscope ISH, β-glucuronidase (GUS) and luciferase (LUC) assays, EMSA and Y1H assays; *ic1* mutant plants showing insensitivity in BR signaling by eBL and PCZ treatment, western blot, quantitative real-time PCR and ectopic overexpression transformation of *Arabidopsis* are described in the *SI Appendix* and *SI Appendix*, *Materials and Methods*.

## Supplementary Material

Supplementary File

## Data Availability

All of the study data are included in the article and/or supporting information.
