# Supplementary material for: OCTOPUS regulates BIN2 to control leaf curvature in Chinese cabbage
Source: Proc Natl Acad Sci U S A. 2022 Aug 15;119(34):e2208978119. doi: 10.1073/pnas.2208978119 (PMC9407555; doi:10.1073/pnas.2208978119)
Supplement: Supplementary File [file pnas.2208978119.sapp.pdf]

Supporting Information (SI Appendix)

OCTOPUS Regulates BIN2 to Control Leaf Curvature in Chinese Cabbage

Xiaomeng Zhang#, Wei Ma#, Mengyang Liu#, Xing Li, Jingrui Li, Yin Lu, Guanghuan Li, Shu Zhang, Daling Feng, Yanhua Wang, Hao Liang, Shuangxia Luo, Na Li, Aixia Gu, Shuxin Xuan, Xueping Chen, Shuxing Shen*, Jianjun Zhao*

#These authors contributed equally to this work.

Correspondence author: Jianjun Zhao (jjz1971@aliyun.com) and Shuxing Shen (shensx@hebau.edu.cn)

This PDF file includes:

Supplementary text- Materials and Methods

Figs. S1 to S25

Tables S1 to S11

References for SI reference citations

Supplementary Information Text

Materials and Methods

Plant Materials

A mutant library of Chinese cabbage was created *via* EMS mutagenesis on the WT A03 doubled haploid line that has an outward-curling leafy head phenotype (1). A mutant with leaves curving inward at the top of the head (*ic1*) was isolated from the M_6 generation. F_1 and F_2 were developed from the cross between A03 and the *ic1* mutant and were used for genetic analysis of the leafy head shape. An inbred line 85-1 with overlapping leaves on the head was hybridized with *ic1* additional F_1 and F_2 populations for mapping the trait. Two inbred lines (17Q398, 17Q430) with inward curling leaves and two inbred lines (17Q373, 17Q402) with outward curling leaves were used to sequence and check the mutation sites in *BrOPS*. The plants were grown in a plastic tunnel on the experimental farm at Hebei Agricultural University in Baoding (115.47 E, 38.87 N), China.

In August 2017, 60 plants each of A03 and *ic1* of the M_6 generation were grown in the same plastic tunnel at Hebei Agricultural University. At the early heading stage (80 days after sowing), the 16th leaf from the exterior of the developing head was sampled at three sections: apical, middle and bottom sections of the leaf, which are referred to as sections a, b and c, respectively (***SI Appendix*, Fig. S7*A***). At least three biological

replicates were used for each section. All leaf samples were immediately frozen in liquid nitrogen and stored at −80°C for RNA analysis.

Inheritance of the Mutant Trait

The mutant *ic1*, WT, ten F_1 lines, 110 F_2 lines were grown and phenotyped in August 2017. To confirm the mutant trait inheritance results, ten F_1 lines, 276 F_2 plants and their parents were planted in a plastic tunnel in August 2018. The number of plants with inward and outward curling leaves was counted and a Chi-square test was performed. The morphological characteristics of plants were investigated for A03 and *ic1* at different developmental stages.

Candidate Mutant Genes Mapping by MutMap and KASP

Using fresh leaves from 32 F_2 lines with an inward curved phenotype and 30 WT individuals from the cross between WT and the *ic1* mutant, we extracted genomic DNA using the CTAB method, and equal amounts of DNA for each plant were pooled for mutant and WT. The two DNA sample pools were sent to Guangzhou Biologic Biotechnology Co., Ltd. for resequencing using the Illumina HiSeqTM 2500 instrument. Approximately 15 Gb of high-quality read data corresponding to 30× coverage of the genome was obtained for each pooled sample. Low-quality reads, in which > 50% of bases had phred quality scores of ≤ 20, were removed. Filtered reads were aligned to the reference *B. rapa* Chiifu genome (http://brassicadb.org/brad/index.php, v2.0) using BWA (0.7.12)

with the MEM algorithm (2). The WT A03 genome were used as a reference gemomic sequences and SNP analysis was performed on the sequencing results. A SNP index ≥ 0.8 was used to filter the candidate SNP loci.

To verify mutant sites, four individual plants, including two *ic1* plants and two WT plants were selected for gene fragment sequencing. Sequencing primers **(*SI Appendix*, Table S1)** were designed near nonsynonymous SNP or stop-gain mutation sites and four DNA samples were amplified by PCR. The PCR conditions were as follows: forward primer 0.5 μL, reverse primer 0.5 μL, 10× Buffer 1.0 μL, dNTPs 0.8 μL, Taq DNA polymerase 0.5 μL, DNA 1.0 μL (50-1000 ng), and RNase-free ddH_2O to 10 μL. PCR program: 94°C 3 min, 35 cycles of 94°C 45 s, 55°C 30 s, 72°C 45 s, and 72°C 7 min. The PCR products were sequenced by Shanghai Bioengineering Co., Ltd. DNAman software was used to align sequencing results of the WT and *ic1* plants to verify the mutation sites. SNP markers in the mutant gene identified from MutMap (3) were selected for KASP (4) in two WT plants, two *ic1* lines, two F_1 lines and an F_2 (WT × *ic1*) population with 111 individuals. The KASP result was used to analyze the responsible ratio of SNPs for heading types.

Target SSR and SNP-Seq

Using fresh leaves from two plants each for 85-1, *ic1* and their F_1 hybrids and 97 F_2 plants (85-1 × *ic1*), we extracted genomic DNA using the CTAB

method. A DNA library was built and labeled for the DNA of each plant. Eighty-nine accurate single-nucleotide polymorphisms (SNPs) and 58 perfect simple sequence repeats (SSRs) with stable motifs and flanking sequences that are both well-known genome-wide in Chinese cabbage were used to map the loci closely associated with heading types via target SSR-Seq (5) and target SNP-Seq (6).

RNA-Seq Analysis

Total RNA of leaf sections was extracted using the TRIzol reagent (Invitrogen) according to the manufacturer's instructions. RNA purity and concentration were assessed using NanoPhotometer® spectrophotometer (IMPLEN) and the Qubit® RNA Assay Kit in a Qubit 2.0 Fluorometer (Life Technologies). The cDNA library was prepared and sequenced as previously described (7, 8). RNA-Seq data analysis was conducted according to previously described methods (7).

Quantitative Real-Time PCR

A 1-μg aliquot of total RNA for each sample was used for reverse transcription and first-strand cDNA synthesis using the PrimeScript™ RT reagent Kit with gDNA Eraser (TAKARA). SYBR Green Master Mix (Vazyme) was used in qRT-PCR analyses. qRT-PCR analyses was performed in three biological replicates with three technical replicates in the LightCycler® 96 (Roche) under the following conditions: 10 min at 95°C, 40 cycles of 10 s at 95°C, 10 s at 57°C, and 10 s at 72°C. After PCR,

a melting curve was generated by 10 s at 95℃, 60 s at 60℃, and 1 s at 97℃. The $2^{-\Delta Ct}$ × 1000 method was used to calculate relative gene expression levels between WT and *ic1* plants. A Chinese cabbage *actin7* gene (BraA10000789) was used as an internal reference. Gene-specific primers for qRT-PCR are presented in ***SI Appendix*, Table S2**.

Brassinosteroid and Propiconazole (PCZ) Treatment

Thirty plants each of WT and *ic1* mutant were sown on MS medium with different concentrations of 24 epi-brassinolide (eBL, Solarbio) ranging from 1 nM to 1000 nM (1nM, 10nM, 100nM, 1000 nM) dissolved in DMSO, respectively. Plants were grown under long-day conditions in culture bottles (16 h light/8 h dark cycle, 50% humidity, 25°C day and 18°C night). Thirty plants each of WT and *ic1* mutant were sown on MS medium with four concentrations of PCZ (0, 0.1, 1, 5 μM) under the dark condition (50% humidity, 25°C), respectively. The lengths of the roots and hypocotyls were measured after seven days.

Cloning and Plasmid Construction

BrOPS, *Br*m*OPS* and *BrOPS1* were amplified via reverse transcription-PCR (RT-PCR) with A03 and *ic1* cDNA as template using a high-fidelity DNA-polymerase (KOD -Plus- Neo, TOYOBO), successively cloned in a pMD™19-T vector using a cloning kit (TaKaRa, Japan), sequenced, and then transferred to pMDC43 for subcellular localization of BrOPS and BrmOPS with a GFP tag, and pGWB414 for BiFC experimentation in *N.*

benthamiana leaves. *BrOPS* was cloned into pCAMBIA1300-cLUC for split-luciferase complementation assay. *BrBIN2* (BraA01001098) was cloned as described above and transferred to pHB for subcellular localization of BrBIN2 with an RFP tag, pGWB414 for BiFC experimentation, and pCAMBIA1300-nLUC for split-luciferase complementation assay in *N. benthamiana* leaves. BrOPS with a FLAG tag and BrBIN2 with His tag were transferred to pD2P for pull-down assay. AtPIP2A, a PLASMA MEMBRANE INTRINSIC PROTEIN 2A (9), fused to RFP was transferred in pHB as a marker for membrane protein colocalization in *N. benthamiana* leaves. AtWRKY71 (10), fused to CFP was transferred in pHB as a marker for nucleaus protein colocalization. Primers for *BrOPS*, *Br*m*OPS* and *BrBIN2* were designed as listed **(*SI Appendix*, Table S4)**. Promotors of *BrKNAT4.1* (1305bp), *BrKNAT4.2* (1854bp), *BrAS1* (1341bp) were amplified using KOD -Plus- Neo, and then cloned into pBI121 for GUS staining assay, and pAbAi for Y1H assay. *BrBES1*, *BrBES1.2*, *BrBES1.3* were cloned into pCAMBIA1302 for GUS staining assay and into pGADT7 for Y1H assay. BrBES1 with His tag were transferred to pD2P for EMSA assay.

Transient Expression in *N. benthamiana* Leaves

Leaves of 5- to 6-week-old plants of *N. benthamiana* were transformed by coinfiltration of Agrobacterium strains carrying the indicated constructs

according to previously described methods (11). Fluorescence was detected 3 days after infiltration by confocal microscopy.

Split-Ubiquitin Membrane Yeast Two-Hybrid Assay

The direct interaction protein of BrOPS was screened from the NubG-fused cDNA library of Chinese cabbage via the split-ubiquitin membrane yeast two-hybrid assay, according to the user manual from Dualsystem Biotech. PCR-amplified *BrOPS* and *BrmOPS* fragments were inserted into the vector using the unique SfiI restriction site of pDHB1 (Dualsystem Biotech), resulting in two different bait vectors encoding fusion proteins consisting of the bait proteins upstream of the C-terminal Cub-LexA-VP16 fusion partner. The inserts were confirmed to be in-frame with the C-terminal Cub-LexA-VP16 and to be free of mutations by sequencing. A NubG-fused cDNA library was inserted into the vector pPR3-N (Dualsystem Biotech) to screen the interaction partners of BrOPS. To confirm the interaction between BrOPS and BrBIN2, PCR-amplified *BrBIN2* fragment was inserted into the vector using the unique SfiI restriction site of pPR3-N (Dualsystem Biotech), resulting in the prey vector encoding fusion proteins consisting of the prey protein downstream of the mutated N-terminal half of ubiquitin (NubG) fusion partner to confirm the interaction result. The insert was confirmed to be in-frame with the NubG and to be free of mutations by sequencing. The NMY51 (Dualsystem Biotech) yeast strain was transformed by thermal shock,

selected on SD/-Leu/-Trp (DDO) medium and then transferred to SD/-Ade/-His/-Leu/-Trp (QDO) supplemented with increasing concentrations of aureobasidin A (AbA).

β-glucuronidase (GUS) Assay

GUS staining was detected as previously described (12). Quantitative GUS activity assay was measured as described (13).

Safranin-Fast Green Staining

Histological study was performed as described (14). Hypocotyl, root and leaf of 7-day-old WT and *ic1* seedlings, stem and leaf of WT and *ic1* plants at early heading stage were fixed with formalin-acetic acid-alcohol (FAA) with 5% formalin, 5% acetic acid and 90% ethanol (50%). After dehydration through a graded series of ethanol (30, 50, 70, 83, 95, 100, 100%; 1h for each) and transparency via xylene (20, 40, 60, 80, 100, 100% mixed with ethanol; 1.5h for each), samples were embedded in paraffin (Sigma, USA). Sections were cut with an automatic paraffin slicer (Leica NANOCUT) and stained with safranin-fast green double staining (15). After staining, cellulose and some cytoplasm are stained blue or green, and lignified cell walls and nuclei are observed red, which was imaged using a image scanner (Leica VESA8).

Electrophoretic Mobility Shift Assay (EMSA)

The EMSA was performed mainly according to previously described (16). The probes (60bp) for *BrAS1* promoter were marked with biotin. His-

BrBES1 and His alone were expressed in a *Eukaryotic Pichia pastoris* cell-free protein synthesis system (17). Each binding reactions were incubated at 25 °C for 15 min. The incubated samples were loaded into a native 4% polyacrylamide gel and blotted onto a nylon membrane under UV crosslinking. The labeled signals on the gel was detected using a charge-coupled device (CCD) camera.

Yeast One-Hybrid (Y1H) Assay

The Y1H was conducted mainly according to the user manual (Clotech). The Y1HGold yeast strain was cotransformed with pAbAi-*BrKNAT4.1*pro/*BrKNAT4.2* pro/*BrAS1*pro and pGADT7-*BrBES1/ BrBES1.2/ BrBES1.3* by thermal shock, selected on SD/-Leu medium and then transferred to SD/-Leu supplemented with increasing concentrations of aureobasidin A (AbA).

Pull-down

Pull-down assay was carried out using recombinant Flag-BrOPS and His-BrBIN2 expressed and purified from a *Eukaryotic Pichia pastoris* cell-free protein synthesis system (17). The agarose beads were collected by centrifugation and washed three times with equilibration buffer (50 mM Tris, 150 mM NaCl, pH 8.0). Protein samples eluted from the beads, followed by Western blotting analysis with anti-Flag (Abcam) and anti-His (Abcam) antibodies.

LUC assay

The constructors, cLUC-BrOPS and nLUC-BrBIN2 were transferred into *Agrobactium tumefaciens* (strain GV3101) and transiently transformed into *N. benthamiana* leaf epidermal cells. The tobaccos were then cultivated for three days in the dark. Luciferin spray (100 μM; Sigma, 103404-75-7) was smeared on the back of the leaves and retained in dark condition for 5 min before the observation of fluorescence. Images were taken with a low-light cooled charge-coupled device imaging apparatus (Tanon 5200). FTL9 and FD1 were used as positive controls (18). Three biological replicates were conducted in all experiments.

Firefly and Renilla luciferases were measured according to primary described (19). The Promotor of *BrAS1* was cloned into pGreenII 0800-LUC (reporter), and then transformed to *Agrobactium tumefaciens* (strain GV3101) together with pSoup-P19 helper plasmid (19) by electric shock. *BrBES1* was cloned into pCAMBIA1302 (effector), and then transformed to GV3101 by thermal shock. The effector and reporter were mixed as 9:1 (V:V) and transiently transformed into *N. benthamiana* leaf epidermal cells. The tobaccos were cultivated for eight hours in dark and then 72 hours under condition (16h/8h, light/dark). Firefly and Renilla luciferases were measured as the kit manual using a dual-luciferase reporter system (Promega).

RNAscope in situ Hybridization (ISH)

RNAscope was conducted as primarily described (20). Briefly, specific antisense and sense probes of *BrOPS* and *BrAS1* was designed and generated by direct polynucleotide synthesis. Paraffin sections of the WT leaves at the early heading stage were used to hybridization of *BrOPS* and *BrAS1* mRNA, and then were stained according to the manual (RNAscope® 2.5HD).

Western Blot

Total protein from 300mg sample was extracted using 10% (v/v) trichloroacetic acid TCA, and dissolved in SDS sample buffer (60Mm Tris-HCl, pH6.8, 2% (w/v) SDS, 10% (v/v) glycerol, 5% (v/v) β-mercaptoethanol, 0.01% (w/v) bromphenol blue). Total extracts were denaturated by heating at 95°C for 5 min. Cell debris were then pelleted by centrifugation at 12,000rpm for 3 min. Approximately 300mg tobacco leaf sample was used for the nuclei, cytosol and plasma membrane isolation with the MinuteTM Plasma Membrane Protein Isolation Kit (Invent Biotechnologies, Inc., Plymouth, MN, USA; Catalog number: SM-005-P) according to the manufacturer's instructions. The isolated nuclei and membrane samples were then dissolved in 2xSDS loading buffer, and cytosol protein was extracted using the 10% TCA method. The proteins were transferred onto PVDF membranes (Millipore). The membranes were blocked with 5% (w/v) non-fat dry milk in Tris-buffered saline (TBS)/Tween20. Subsequently the membranes were probed 2 hours at

room temperature with a rabbit anti-BES1/BZR1 antibody (PHYTOAB) or with a mouse anti-RFP antibody (Bioss)/ anti-Actin antibody (Engibody). After washing, the membranes were incubated with anti-rabbit or anti-mouse secondary antibody conjugated to HRP (Abclonal). The actin abundance is used as a control.

Concentration Analysis of Brassinobide (BL)

The concentration of BL was analyzed using a high-pressure liquid chromatography-electrospray tandem mass spectrometry (LC-ESI-MS/MS) system. Three technical replicates and three biological replicates were conducted.

Fluorescence Intensity Measured by ImageJ

The fluorescence or chemiluminescence intensity in confocal and western blot assays was measured using ImageJ software (21). Images were changed to 8-bit type; mean gray value, min/max gray value, and integrated density were measured within the measurement area, and only light background was selected. The unit of length was changed to pixels, freehand selection was chosen, and the image was inverted. A bright target sections, including entire nucleus and cell's edges (considered as plasma membrane) portion, were selected as the measurement area, and mean gray value, min/max gray value, and integrated density were measured. The gray integrated density value was used as the measure of fluorescence or chemiluminescence intensity.

Transient Gene Expression in Chinese cabbage protoplasts

BrOPS-GFP, BrmOPS-GFP, BrBIN2-RFP were cloned into pBI221 for subcellular localization in Chinese cabbage protoplasts. AHA1, Arabidopsis plasma membrane H^+-ATPase1 (22), fused to mCherry was transferred in pBI221 as a marker for membrane protein colocalization in protoplasts. Chinese cabbage was sown in pots under a climate chamber (16h light/8h dark, 50% humidity, 25℃ day/18℃ night). The protoplasts were extracted from leaves of healthy two-week-old plants, and transiently transformed with the indicated constructs according to previously described methods (23). Fluorescence was detected one day after infiltration by confocal microscopy.

Agrobacterium-mediated transformation of Arabidopsis

Arabidopsis plants (Col-0 and *bri1-116*) were transformed with *Agrobacterium tumefaciens* (GV3101) by the floral dipping method (24) and transgenic lines carrying GFP-BrOPS were selected on hygromycin (30mg/L) selection medium. For primer sequences for transgenic insert and genotype identification see Supplementary Table S11.

SI Appendix Figures

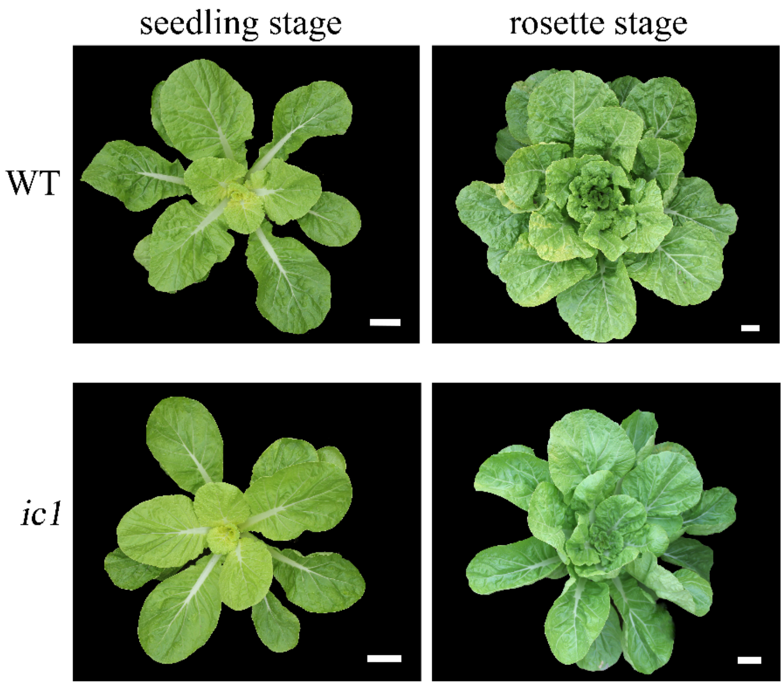

Figure S1. WT and *ic1* plant phenotypes across two developmental stages (seedling and rosette). Bar, 10 cm.

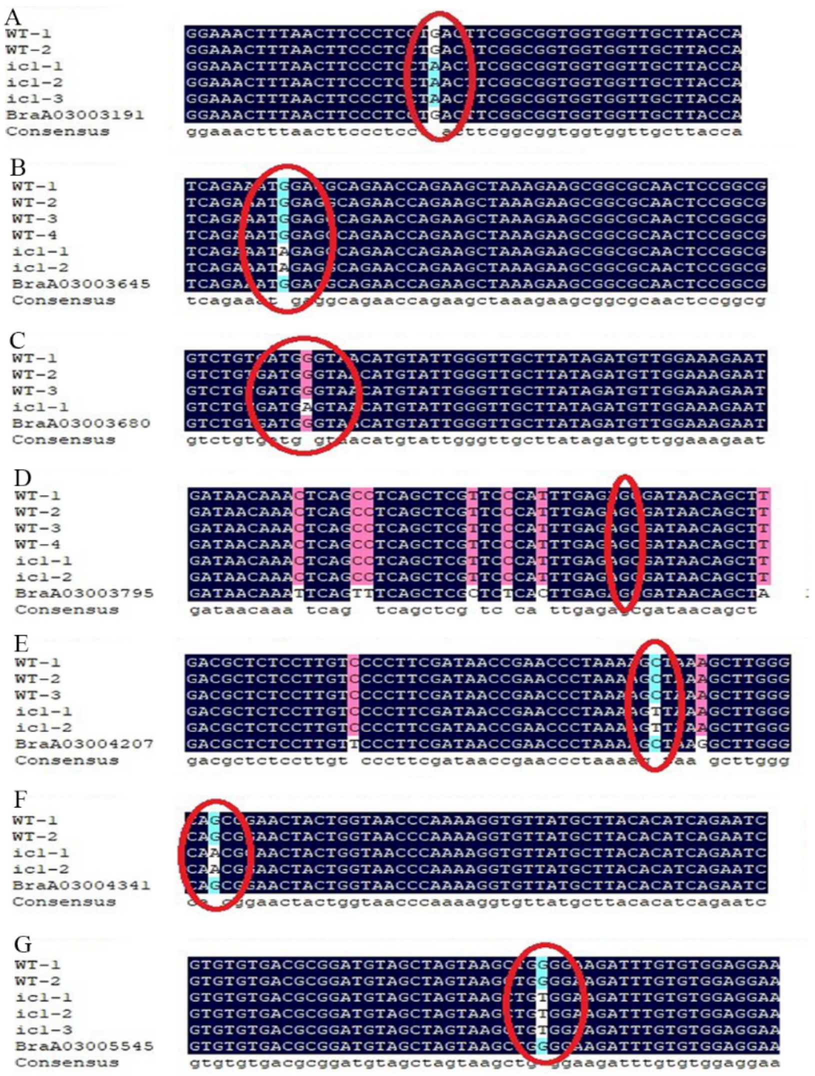

Figure S2. PCR sequence analysis of seven mutant genes identified by MutMap. (*A*) BraA03003191, (*B*) BraA03003645, (*C*) BraA03003680, (*D*) BraA03003795, (*E*) BraA03004207, (*F*) BraA03004341, and (*G*) BraA03005545. Pink indicates at least 90% sequence identity at the mutant sites; blue indicates 50% – 80% sequence identity. The red ovals represent mutant sites identified in MutMap. PCR sequence analysis indicated that there was no mutation in BraA03003795 (*D*).

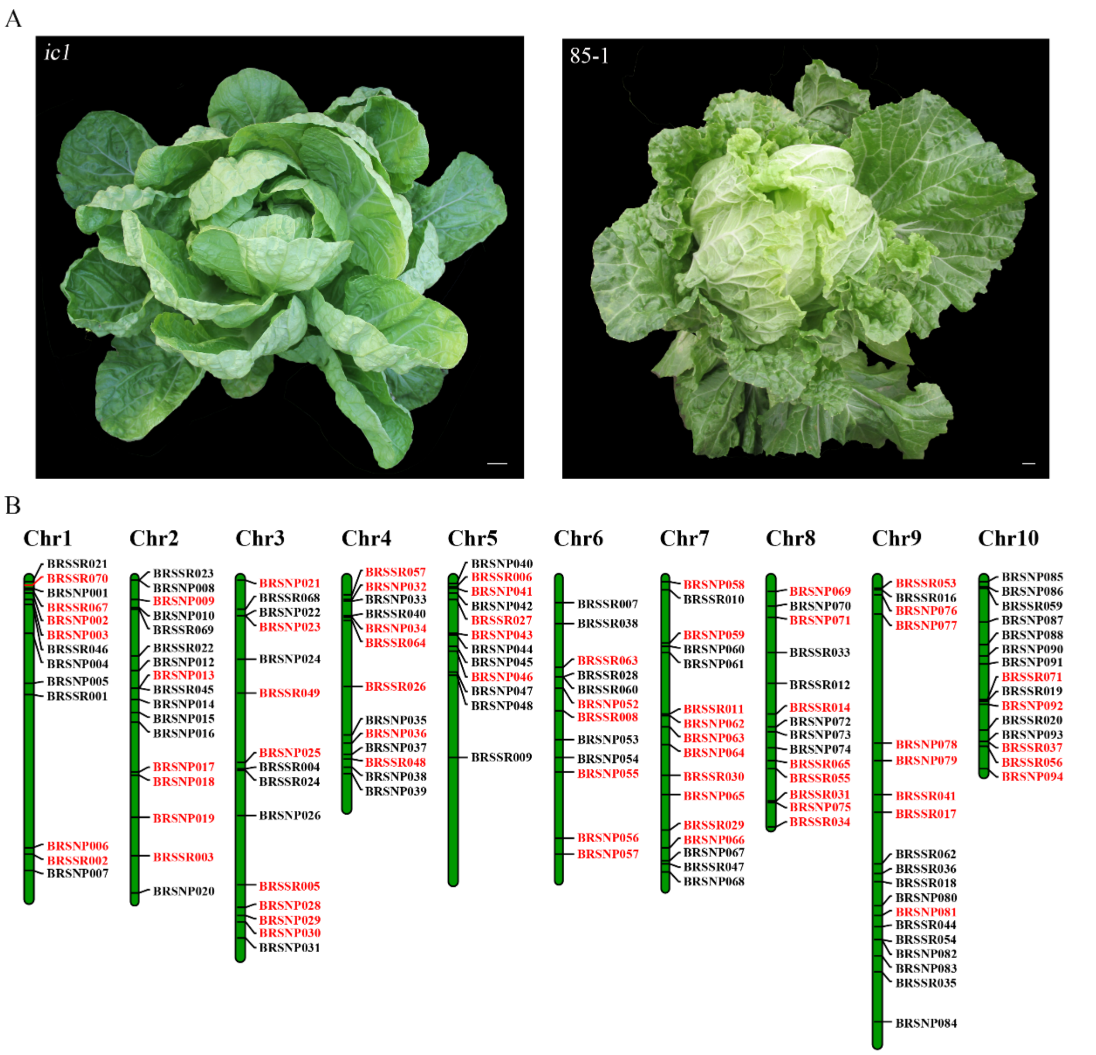

Figure S3. Identification of candidate locus associated with heading type using the Target SNP and SSR-Seq. (*A*) An inbred line 85-1 (overlapping leaves on the head) was crossed with *ic1* (inwardly curled shape without an overlapping leafy head) to create additional F_1 and F_2 populations. (*B*) Locations of 89 SNPs and 58 SSRs on ten chromosomes of Chinese cabbage. Red indicates the 41 SNPs and 28 SSRs identified as polymorphic in F_2 lines (85-1 × *ic1*).

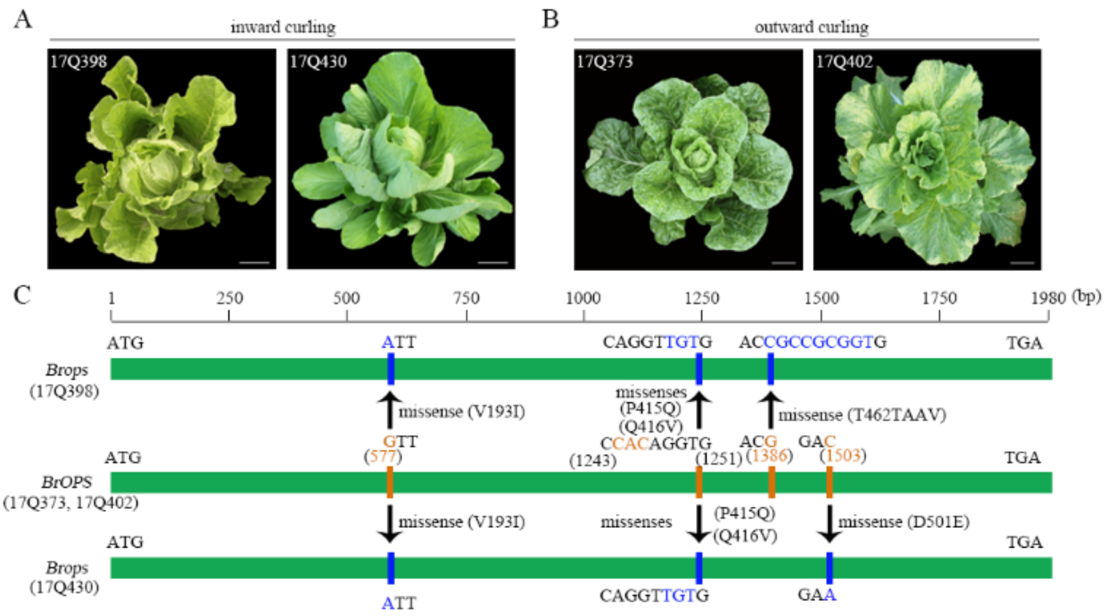

Figure S4. The variation of *BrOPS* in the Chinese cabbage inbred lines (17Q398, 17Q430) with an inwardly curved head and (17Q373, 17Q402) with an outwardly curved head. (*A*) The phenotype of 17Q398 and 17Q430. Bar, 10 cm. (*B*) The phenotype of 17Q373 and 17Q402. Bar, 10 cm. (*C*) Schematic diagram of the *BrOPS* variation in 17Q398, 17Q430, 17Q373 and 17Q402.

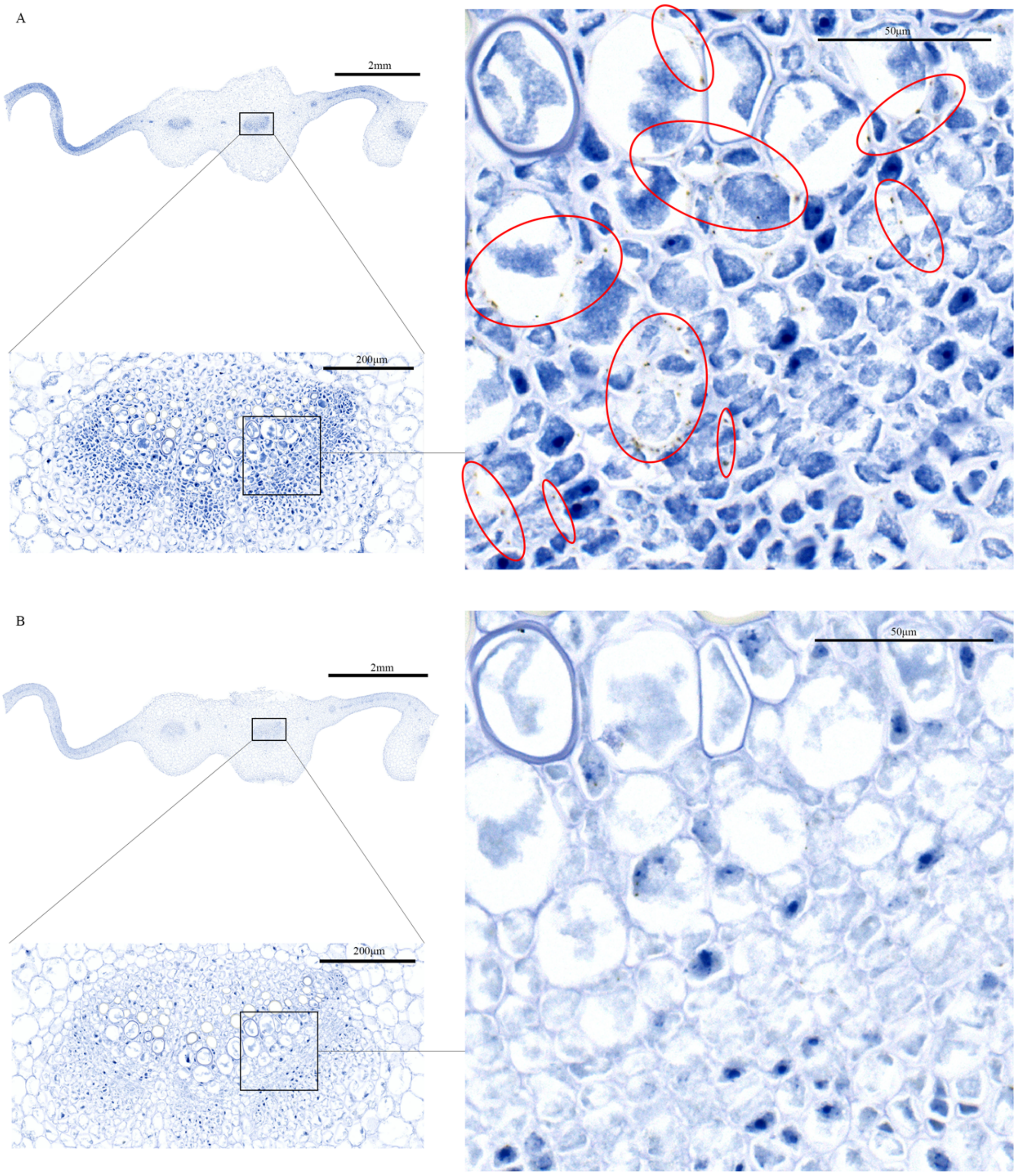

Figure S5. The tissue location of *BrOPS* detected *via* RNAscope ISH at the early heading stage. (*A*) The brown dots within the red circles indicate *BrOPS*. *BrOPS* expression was observed in the meristem between the xylem and phloem, in the xylem parenchyma cells, and in undifferentiated vessel cells. (*B*) *BrOPS* expression detected using the sense probe of *BrOPS via* RNAscope ISH as a negative control.

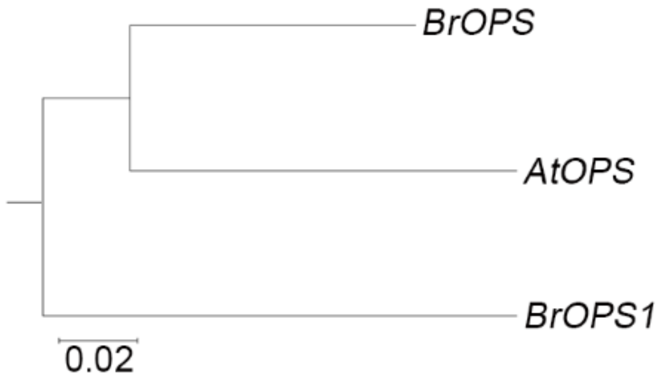

Figure S6. Phylogenetic analysis of nucleic acid sequences of *BrOPS*, *BrOPS1*, and Arabidopsis *OPS* (*AtOPS*).

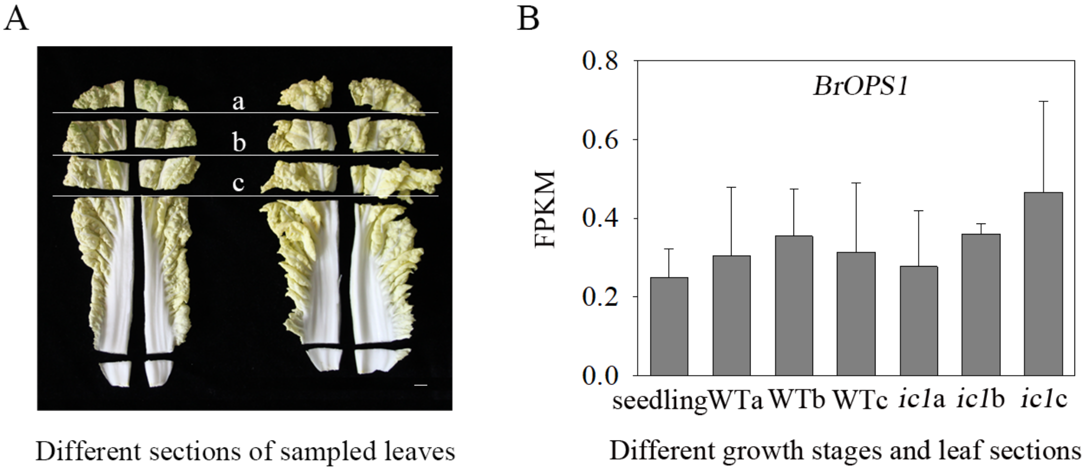

Figure S7. Transcript level of *BrOPS1*. (*A*) Leaf samples at the early heading stage for RNA-Seq and qRT-PCR. The leaf was divided into three sections (a, b, and c). (*B*) The expression level of *BrOPS1* measured by RNA-Seq at the seedling and early heading stage in WT and *ic1* plants. a, b, and c correspond to the three leaf sections shown in (A), n = 3.

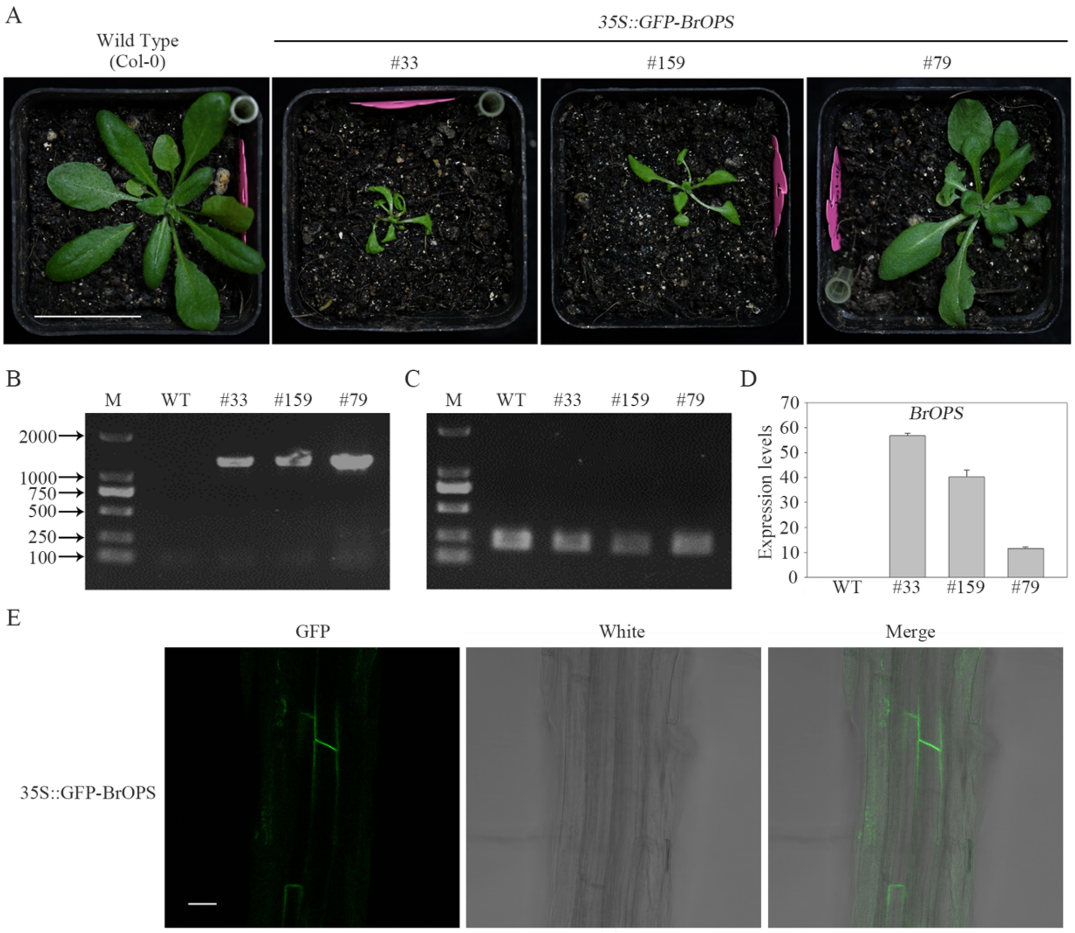

Figure S8. Stable transgenic Col-0 Arabidopsis plants overexpressing *GFP-BrOPS* gene exhibited the outward-curling leaves. (*A*) Altered leaf phenotypes of the plants transgenic for *BrOPS* in three transformed Arabidopsis alleles. Bar, 2 cm. (*B*) The identification of transgenic Arabidopsis plants using PCR. M, DNA marker. (*C*) the genotype of transformed Arabidopsis plants using PME I enzyme digestion. M, DNA marker. (*D*) The transcript levels of *BrOPS* in three transgenic Arabidopsis alleles overexpressing *BrOPS* gene. (*E*) The polar localization of GFP-BrOPS in roots of transformed Arabidopsis. Bar, 20 μm.

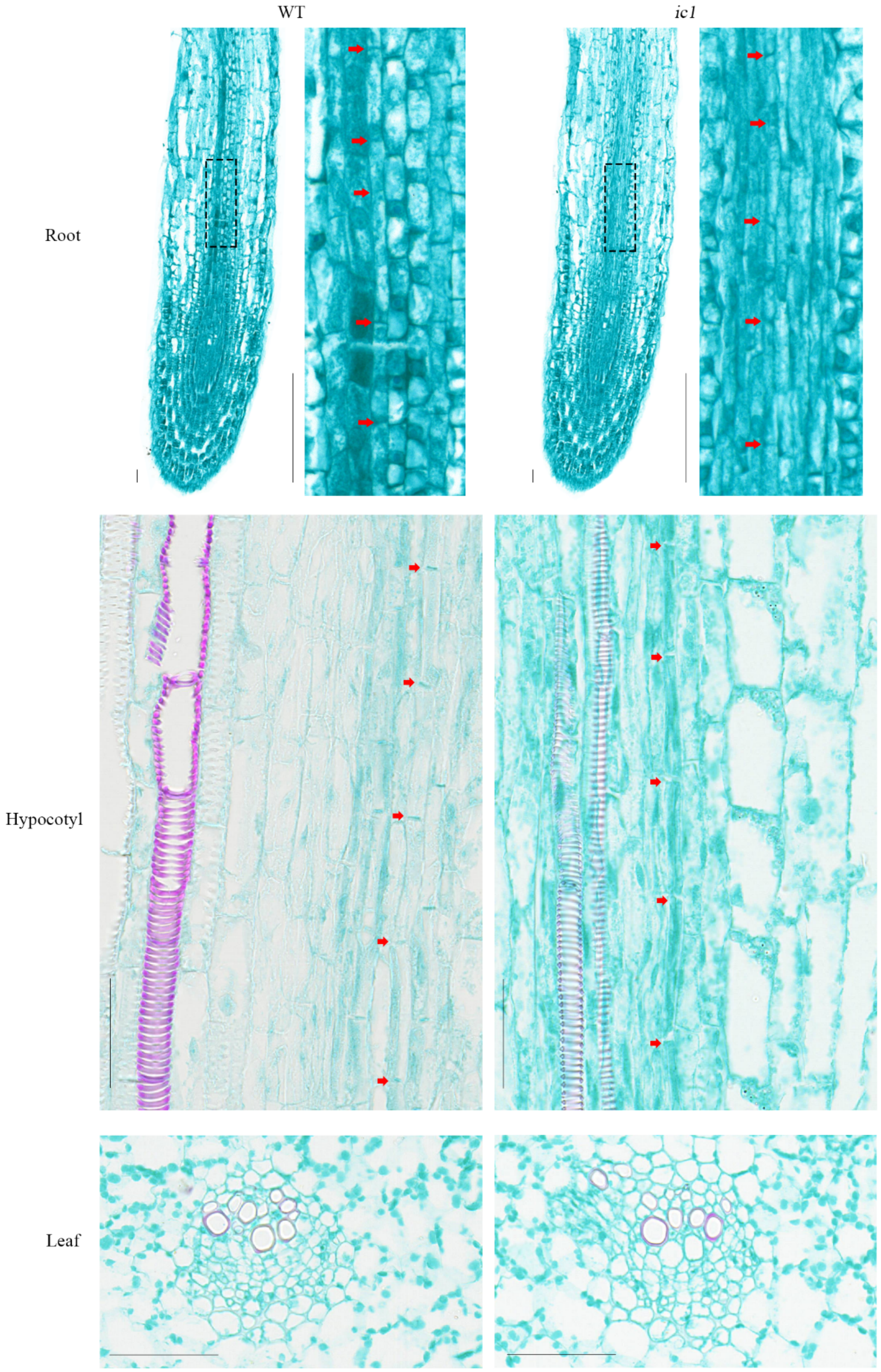

356

Figure S9. Vascular phenotypes in roots, hypocotyls and leaves of 7-day-old WT and *ic1* seedlings stained with safranin–fast green. Red arrows, phloem in hypocotyls and protophloem in roots. Bar, 50 μm.

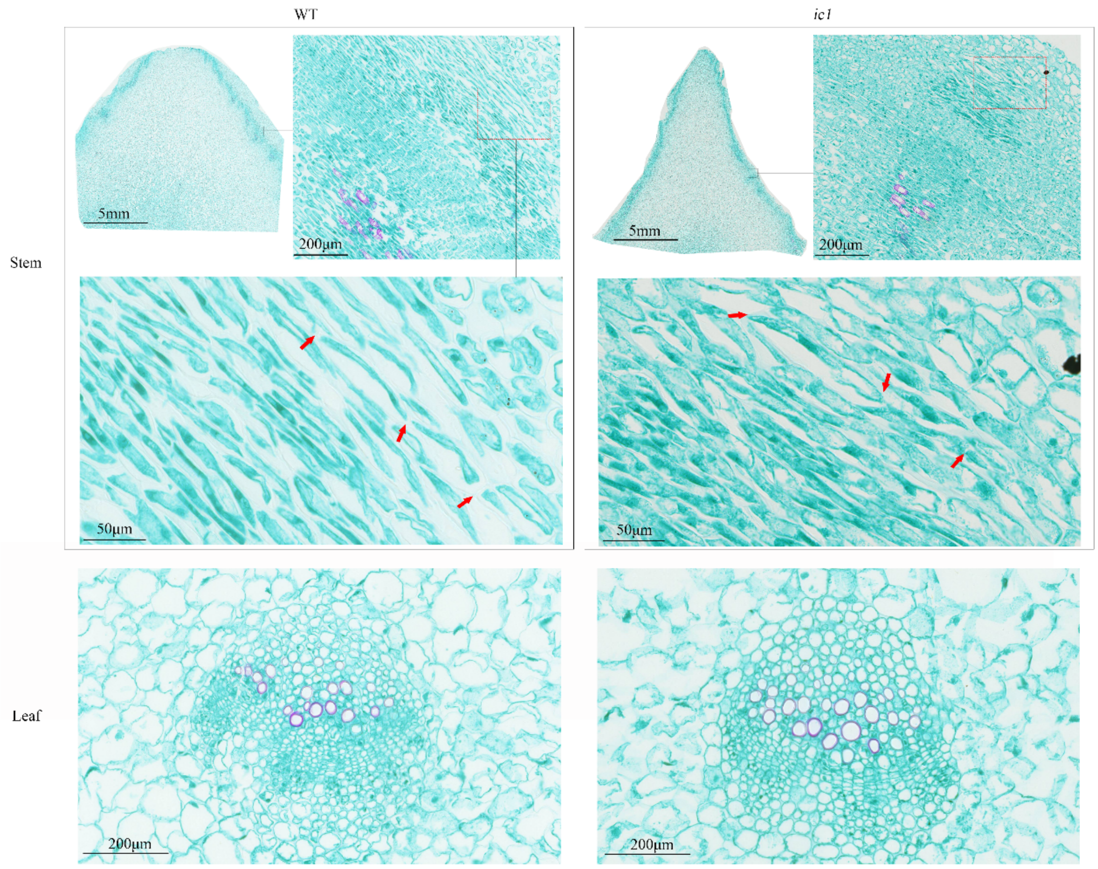

Figure S10. Vascular phenotypes in stems and leaves of WT and *ic1* plants at the early heading stage stained with safranin–fast green. Red arrows, phloem in stems.

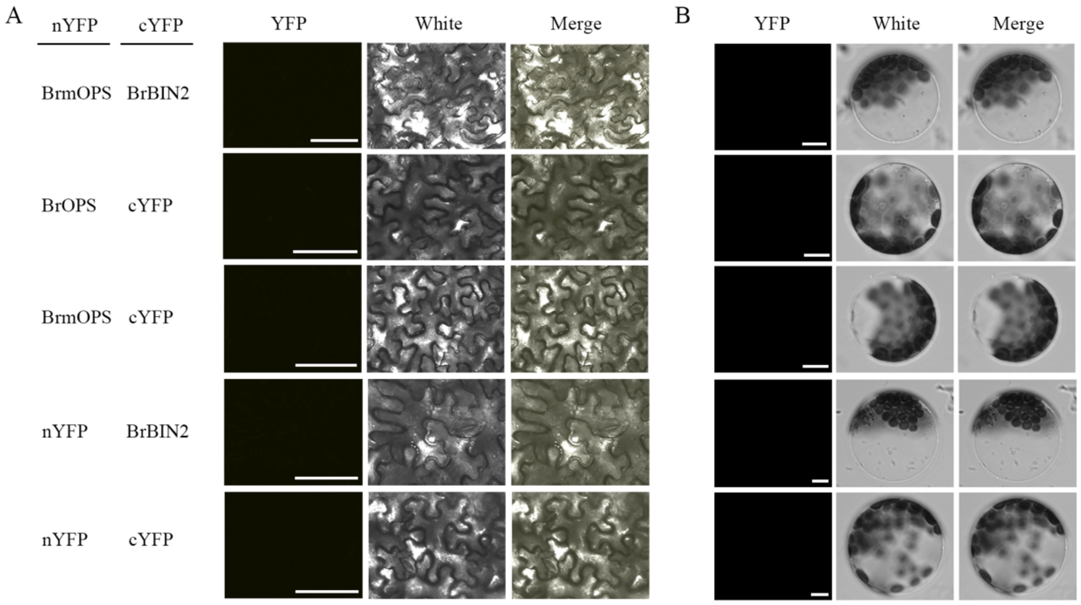

Figure S11. The controls for BrOPS and BrBIN2 interaction visualized in a split-YFP assay by confocal microscopy. (*A*) The controls of BrOPS and BrBIN2 interaction in transiently transformed *N. benthamiana* epidermal cells. Bar, 50 μm. (*B*) The controls of BrOPS and BrBIN2 interaction in transiently transformed Chinese cabbage protoplasts. Bar, 10 μm.

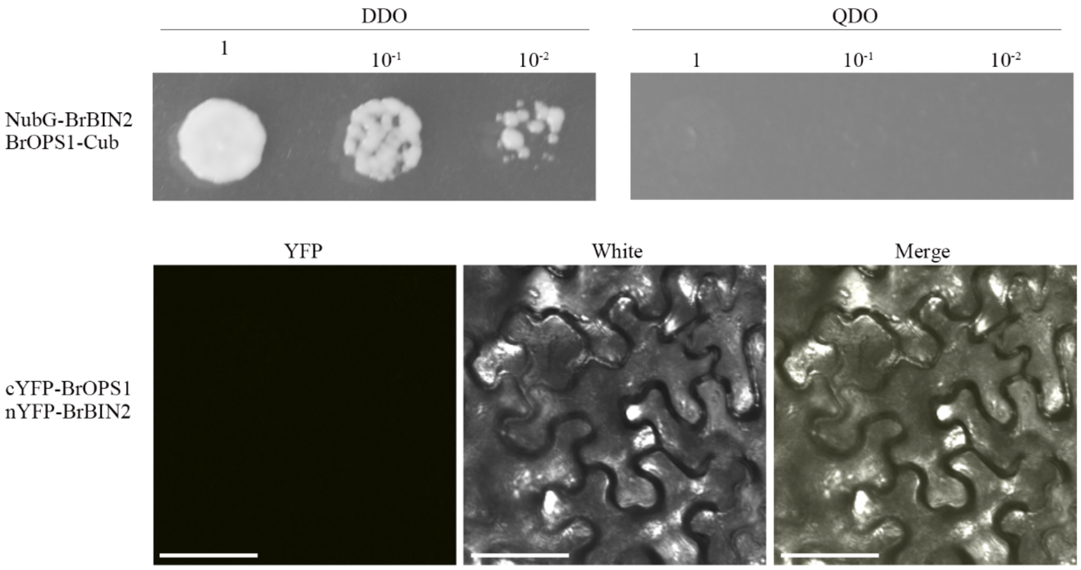

Figure S12. BrOPS1 and BrBIN2 exhibited no interaction in the split-ubiquitin membrane yeast two-hybrid system and the *N. benthamiana* transient expression system. Bar, 50 μm.

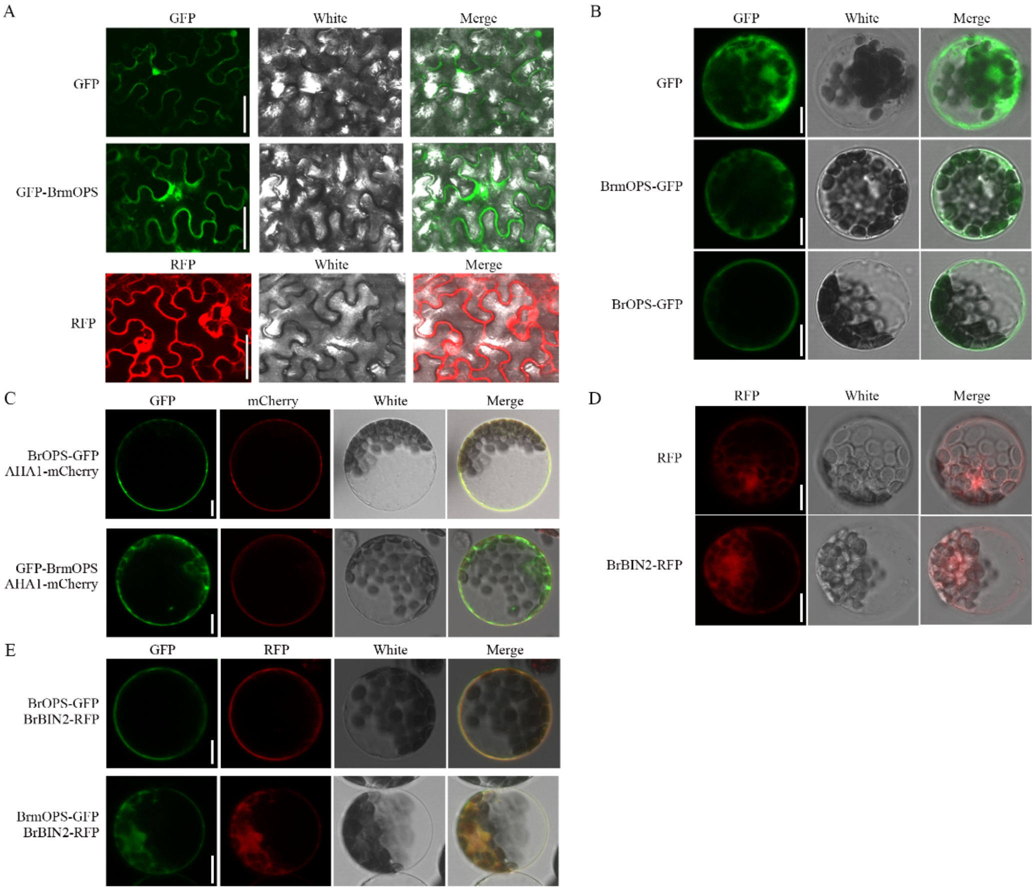

Figure S13. The subcellular localization of BrOPS and BrBIN2. (*A*) Confocal imaging of *N. benthamiana* epidermal cells transiently expressing free-GFP, GFP-BrmOPS and free-RFP. Bar, 50 μm. (*B*) Confocal imaging of Chinese cabbage protoplasts transiently expressing free-GFP, BrmOPS-GFP and BrOPS-GFP. Bar, 10 μm. (*C*) Confocal imaging of Chinese cabbage protoplasts transiently co-expressing BrOPS-GFP/BrmOPS-GFP and the plasma membrane marker protein Arabidopsis P-ATPase1 (AHA1)-mCherry. Bar, 10 μm. (*D*) Confocal imaging of Chinese cabbage protoplasts transiently expressing free-RFP and BrBIN2-RFP. Bar, 10 μm. (*E*) BrBIN2-RFP localization in the presence of

BrOPS/BrmOPS visualized in Chinese cabbage protoplasts by confocal microscopy. Bar, 10 μm.

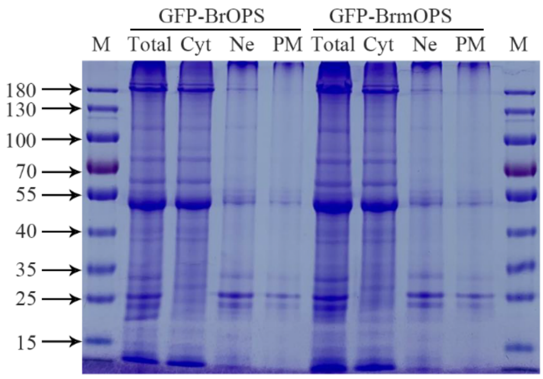

Figure S14. Coomassie brilliant blue staining of total protein (Total), cytosolic protein (Cyt), nuclear protein (Ne) and plasma membrane (PM) from tobacco leaves co-expressing BrBIN2-RFP and GFP-BrOPS/GFP-BrmOPS. M, protein marker.

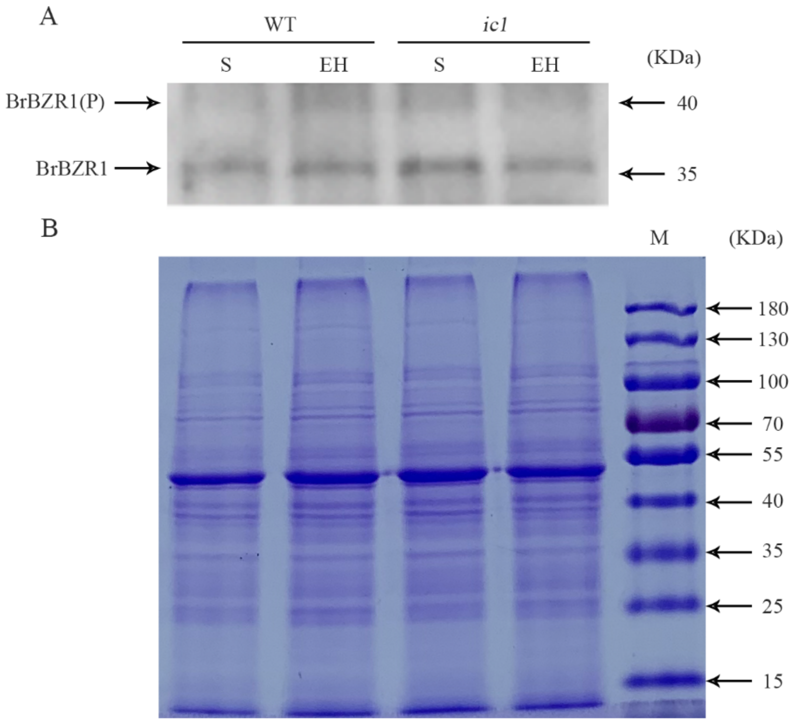

Figure S15. BrBZR1 accumulation levels and phosphorylation status, and coomassie brilliant blue staining of leaf total protein from WT and *ic1* Chinese cabbage at the seedling and early heading stages. (*A*) BrBZR1 abundance and phosphorylation status of leaf total protein from WT and *ic1* Chinese cabbage at the seedling and early heading stages. (*B*) coomassie brilliant blue staining of leaf total protein from WT and *ic1* Chinese cabbage at the seedling and early heading stages. M, protein marker.

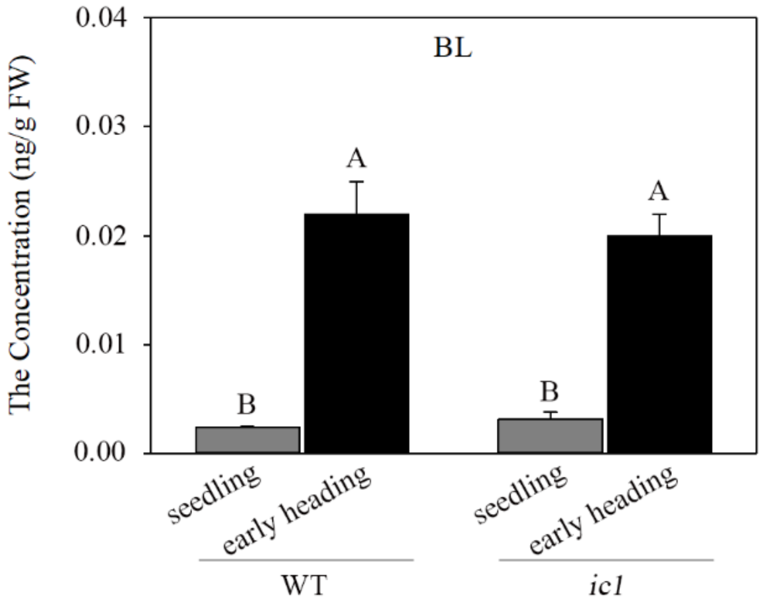

Figure S16. The concentrations of brassinolide (BL) in WT and *ic1* Chinese cabbage at the seedling and early heading stages. Error bars, SD (n = 3). Significance was determined by ANOVA.

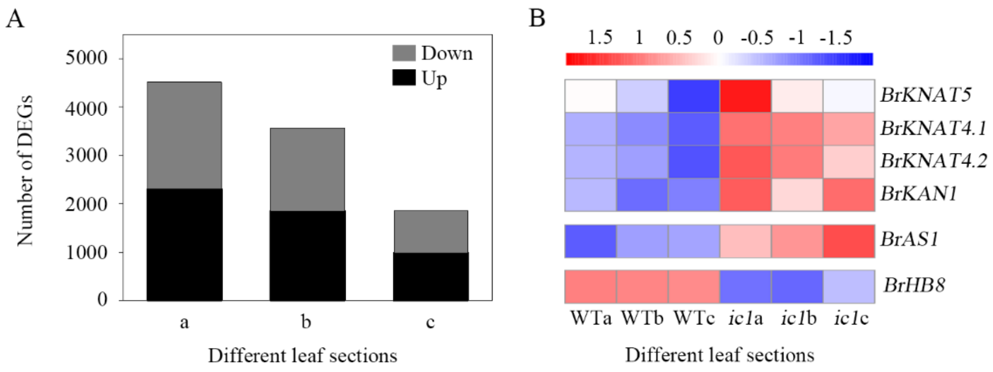

Figure S17. Transcript levels of genes at the early heading stage in *ic1* compared with WT. (*A*) Total number of differentially expressed genes (DEGs) in *ic1* compared with WT measured by RNA-Seq in different leaf sections. (*B*) The heat-map showing the transcript levels of six axial polarity genes measured by RNA-Seq. a, b, and c correspond to the leaf samples shown in Figure. S7*A*.

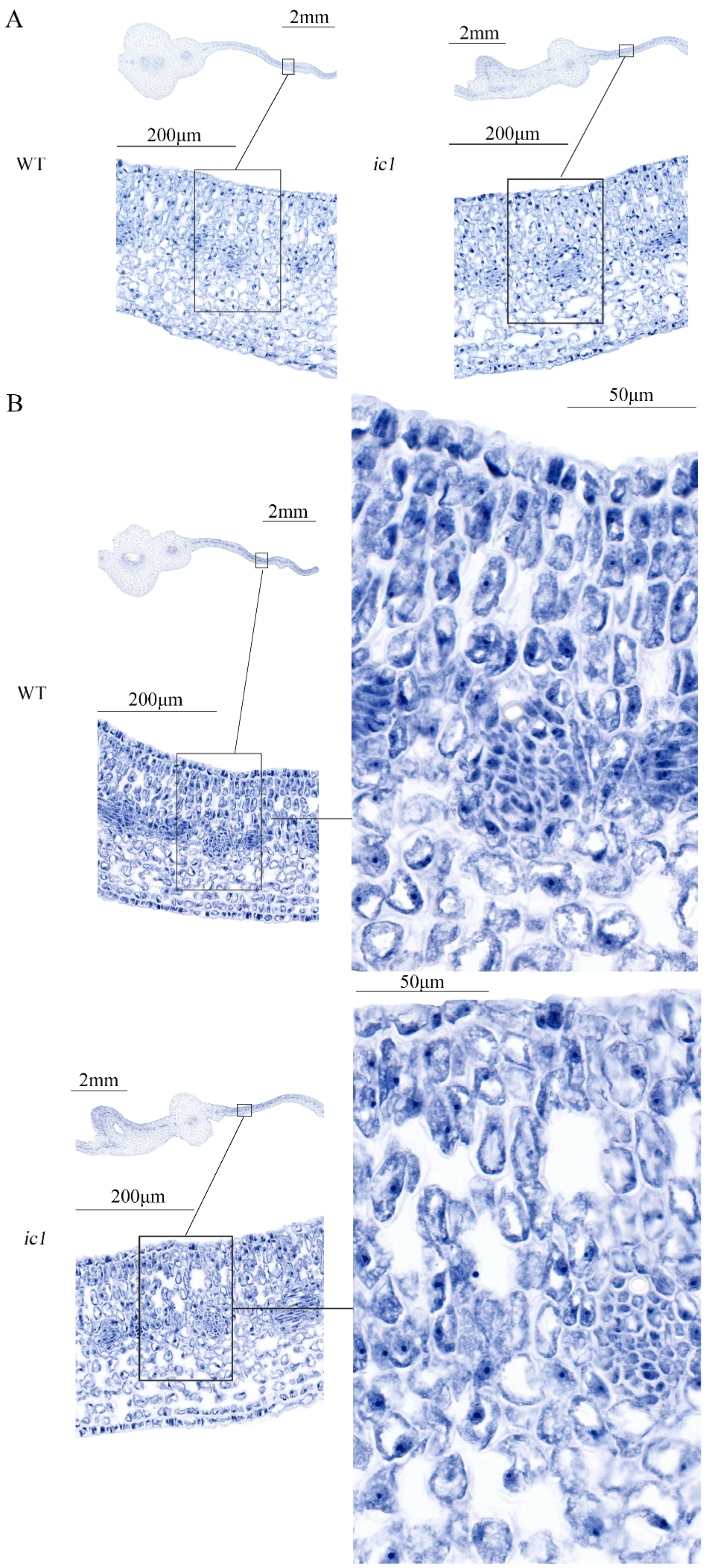
A
2mm
200µm
WT
2mm
200µm
ic1
B
50µm
2mm
WT
200µm
50µm
2mm
200µm
ic1

418

Figure S18. The controls of *BrAS1* tissue expression in WT and *ic1* detected *via* RNAscope ISH at the early heading stage shown in Figure 5*C*. The brown dots in red circles indicate *BrAS1*. (*A*) The full view of WT and *ic1* tissue shown in Figure 5*C*. (*B*) *BrAS1* expression detected in WT and *ic1* using the sense probe of *BrAS1* as a negative control.

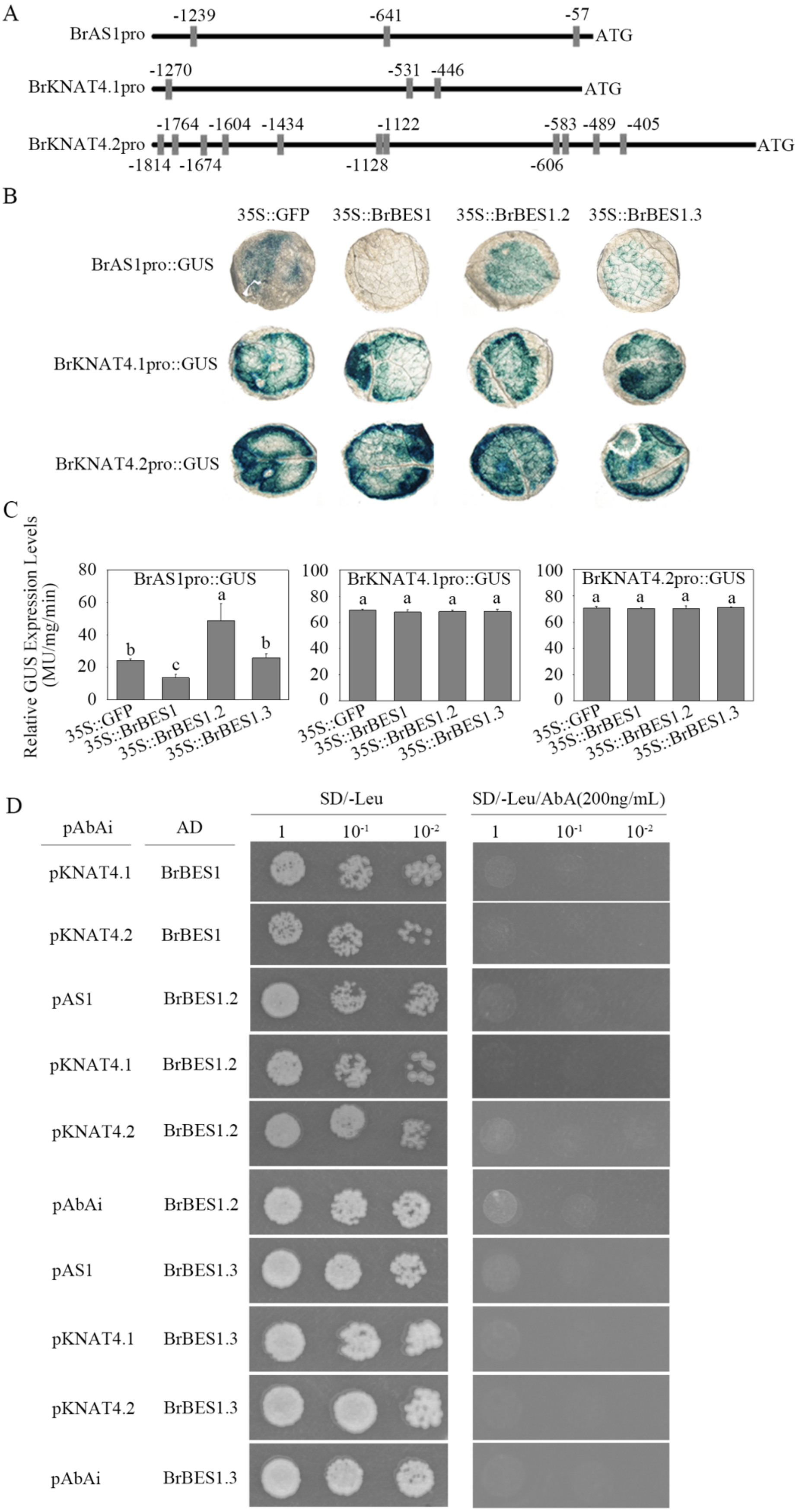
A
BrAS1pro
-1239
-641
-57
ATG
BrKNAT4.1pro
-1270
-531
-446
ATG
BrKNAT4.2pro
-1764
-1604
-1434
-1122
-583
-489
-405
-1814
-1674
-1128
-606
ATG
B
35S::GFP
35S::BrBES1
35S::BrBES1.2
35S::BrBES1.3
BrAS1pro::GUS
BrKNAT4.1pro::GUS
BrKNAT4.2pro::GUS
C
Relative GUS Expression Levels (MU/mg/min)
BrAS1pro::GUS
BrKNAT4.1pro::GUS
BrKNAT4.2pro::GUS
35S::GFP
35S::BrBES1
35S::BrBES1.2
35S::BrBES1.3
D
pAbAi
AD
SD/-Leu
SD/-Leu/AbA(200ng/mL)
1
10-1
10-2
pKNAT4.1 BrBES1
pKNAT4.2 BrBES1
pAS1 BrBES1.2
pKNAT4.1 BrBES1.2
pKNAT4.2 BrBES1.2
pAbAi BrBES1.2
pAS1 BrBES1.3
pKNAT4.1 BrBES1.3
pKNAT4.2 BrBES1.3
pAbAi BrBES1.3

Figure S19. The regulation of BrBES1 on the axial polarity genes *BrKNAT4.1*, *BrKNAT4.2* and *BrAS1*. (*A*) Promoters of the three axial polarity genes *BrKNAT4.1*, *BrKNAT4.2*, and *BrAS1*. Boxes indicate E-boxes (CANNTG). (*B*) BrBES1 represses downstream GUS activity driven by the promoter of *BrAS1* but does not regulate *BrKNAT4.1* or *BrKNAT4.2*. BrBES1.2 and BrBES1.3 are two copies of BrBES1. (*C*) Quantification of GUS activity showing in (*B*). Error bars, SD (n = 5). Significance was determined by ANOVA. (*D*) The detection of BrBES1, BrBES1.2 and BrBES1.3 binding the promoters of *BrAS1*, *BrKNAT4.1* and *BrKNAT4.2* in a yeast one-hybrid (Y1H) assay.

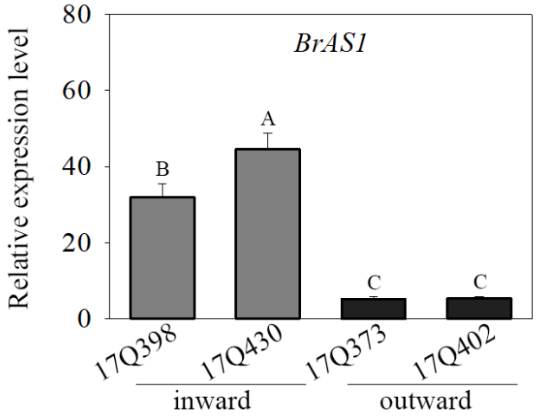

Figure S20. Transcript levels of *BrAS1* in two inward curling inbred lines (17Q398 and 17Q430) and two outward curling inbred lines (17Q373 and 17Q402). Error bars, SD (n = 3). Significance was determined by ANOVA.

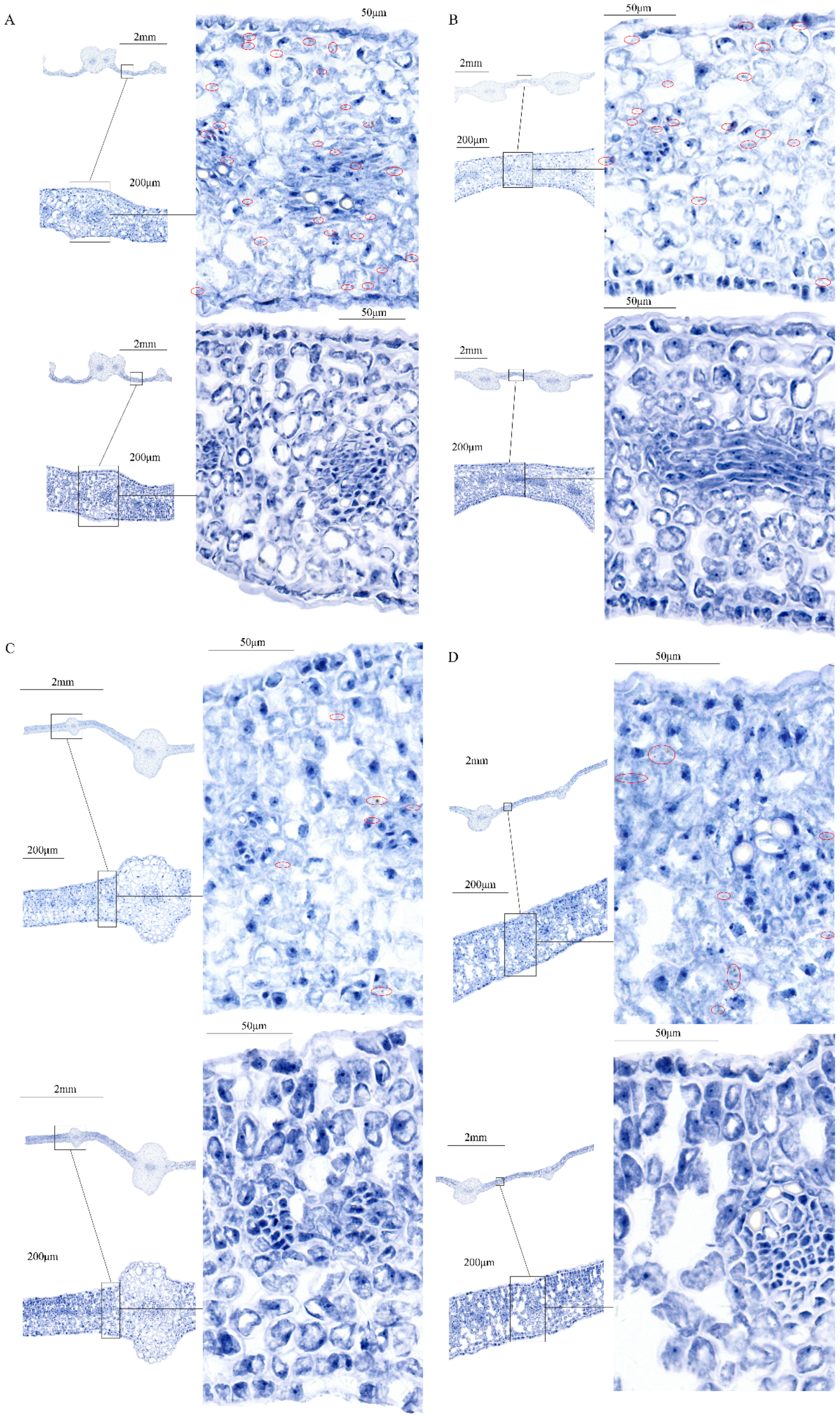

A
B
C
D
2mm
200μm
50μm

Figure S21. The tissue locations of *BrAS1* in two inward curling inbred lines (17Q398 and 17Q430) and two outward curling inbred lines (17Q373 and 17Q402) detected *via* RNAscope ISH at the early heading stage. The brown dots in red circles indicate *BrAS1*. (*A*) The tissue locations of *BrAS1* in 17Q430. The bottom is *BrAS1* expression detected using the sense probe of *BrAS1* as a negative control. (*B*) The tissue locations of *BrAS1* in 17Q398. The bottom is *BrAS1* expression detected using the sense probe of *BrAS1* as a negative control. (*C*) The tissue locations of *BrAS1* in 17Q373. The bottom is *BrAS1* expression detected using the sense probe of *BrAS1* as a negative control. (*D*) The tissue locations of *BrAS1* in 17Q402. The bottom is *BrAS1* expression detected using the sense probe of *BrAS1* as a negative control.

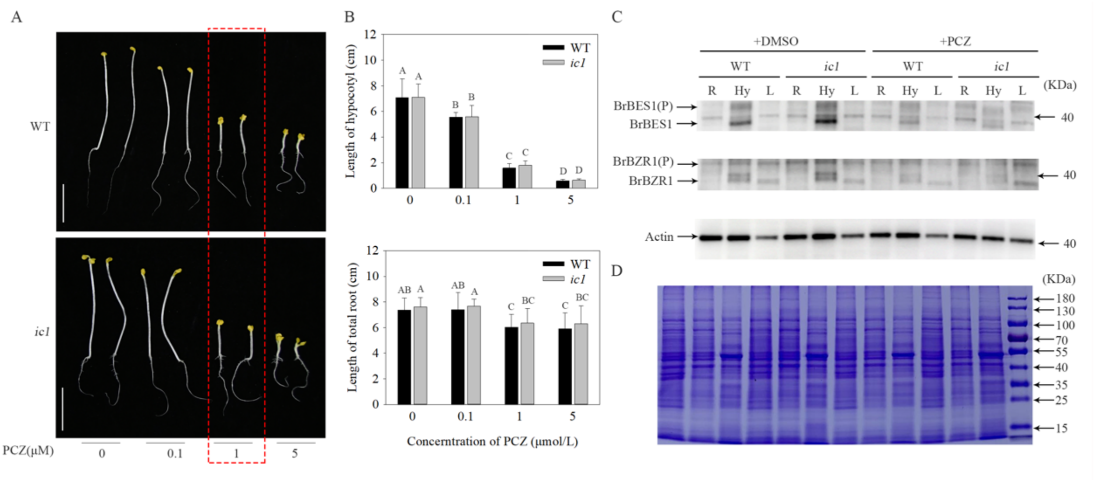

Figure S22. ***ic1*** **mutant exhibited no difference from WT treated with different concentrations of propiconazole (PCZ)**. (*A*) Seven-day-old in WT and *ic1* mutant seedlings grown on Murashige & Skoog (MS) medium with 1% sucrose supplemented with different concentrations of PCZ ranging from 0.1 μM to 5 μM. Bar, 2 cm. The WT and *ic1* mutant seedlings treated with 1 μM PCZ in the red box were used to assess the phosphorylation status of BrBES1 and BrBZR1 in roots, hypocotyls and leaves. Bar, 2 cm. (*B*) Hypocotyl (up) and total root (bottom) length of seedlings shown in (*A*). Error bars, SD (n = 30). Significance was determined by ANOVA. (*C*) The phosphorylation levels of BrBES1 and BrBZR1 in roots, hypocotyls, and leaves of WT and *ic1* seedlings treated with DMSO or PCZ. (*D*) Coomassie brilliant blue staining of total protein in roots, hypocotyls, and leaves of WT and *ic1* seedlings treated with DMSO or PCZ.

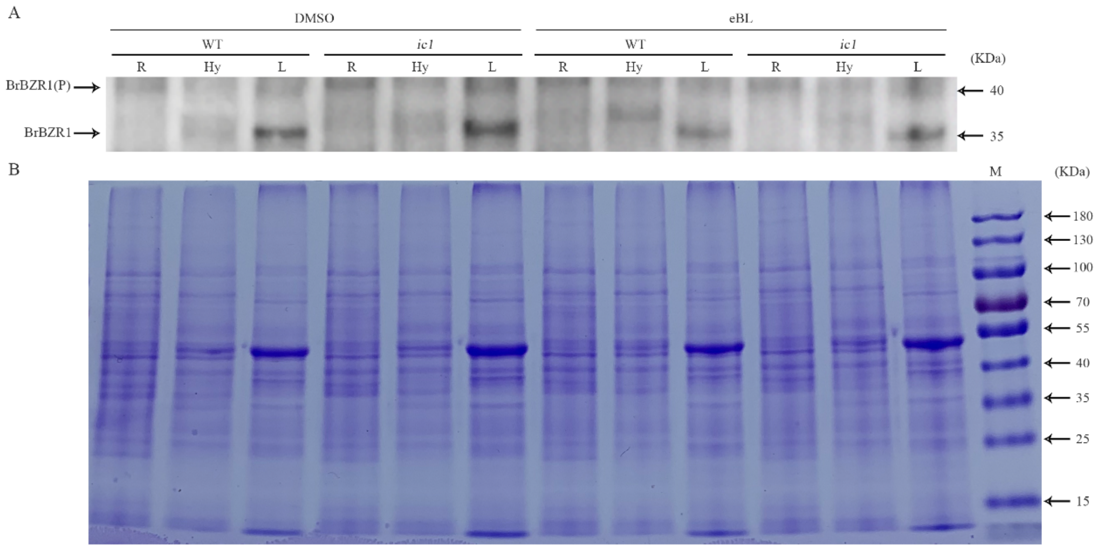

Figure S23. BrBZR1 accumulation levels and phosphorylation status, and coomassie brilliant blue staining of total protein from roots, stems, and leaves of WT and *ic1* Chinese cabbage treated with DMSO and eBL. (*A*) BrBZR1 abundance and phosphorylation status of total protein from roots, stems, and leaves of WT and *ic1* Chinese cabbage treated with DMSO and eBL. (*B*) coomassie brilliant blue staining of total protein from roots, stems, and leaves of WT and *ic1* Chinese cabbage treated with DMSO and eBL. M, protein marker.

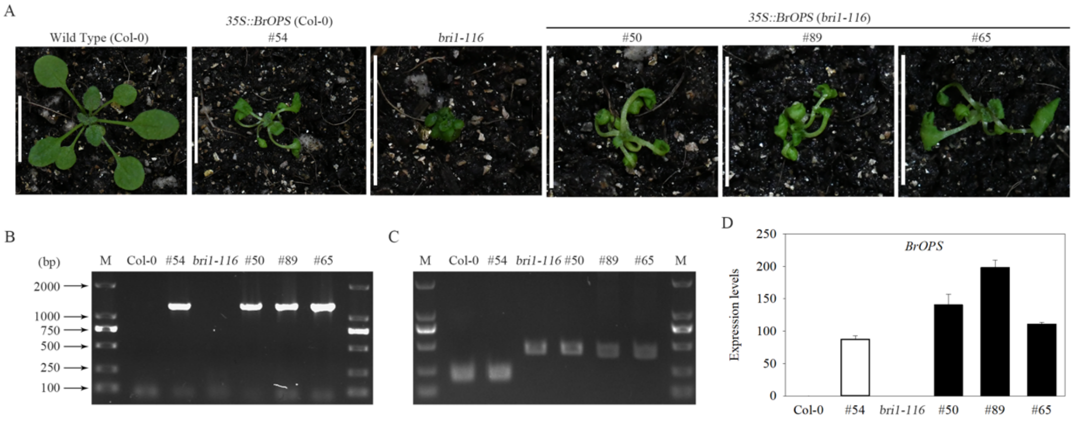

Figure S24. Ectopic overexpression of *BrOPS* in *bri1-116* rescues the *bri1-116* phenotypes and results in outward-curling leaves. (*A*) The outward curling leaf phenotypes of three alleles overexpressing *BrOPS* in *bri1-116*. Bar, 1cm. (*B*) The identification of transgenic Arabidopsis plants using PCR. M, DNA marker. (*C*) The genotype of transformed Arabidopsis plants using PME I enzyme digestion. M, DNA marker. (*D*) The transcript levels of *BrOPS* in transgenic Arabidopsis alleles overexpressing *BrOPS* gene.

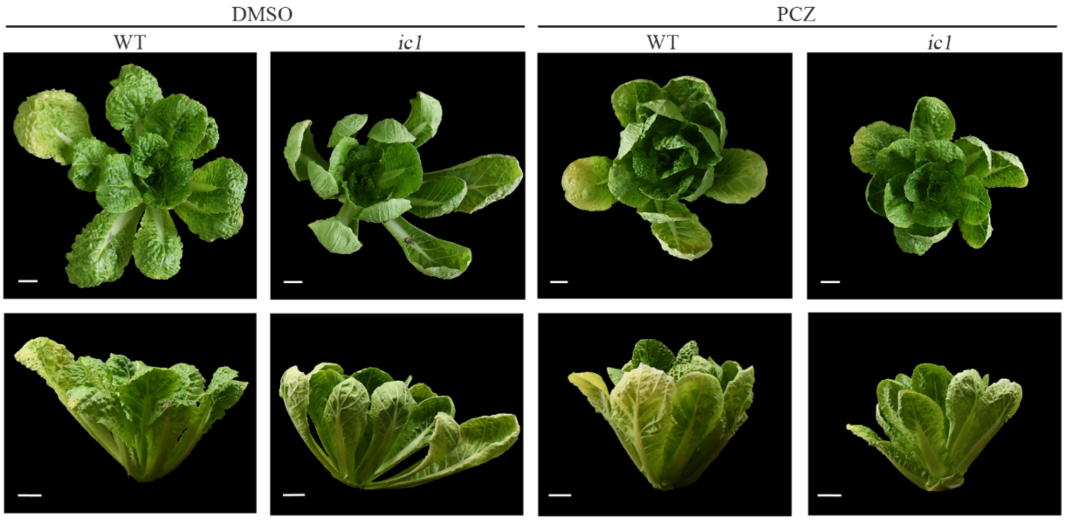

Figure S25. The leaf phenotype of WT and *ic1* after twice 100 μM PCZ application to soil in pots. The newly expanded leaves of PCZ-treated WT plants displayed inward-curling phenotype similar to *ic1* mutant after 30 days with twice 100 μM PCZ treatment at the rosette stage. Bar, 2 cm.

Supplementary Table S1 Primers designed for gene fragment sequencing and cloning

Gene	Forward (5’-3’)	Reverse (5’-3’)
BraA03003191	ACACGAAGCCTGTTTACT	CCATAACGGTTCTTCACT
BraA03003645	GGAAACGCCAACGGTAAC	CGTCGAGCGAGAATCTAG
BraA03003680	TTGGAAGTGCAGAAGCGAC	CATACCCACAAACGAAGGC
BraA03003795	AAAGTGAACGCCCTGTGAA	GGGTGATACGGTCATTGGA
BraA03004207	CATAACCAAACCCAAAGCA	AATCCTCAAACGCAAAGTC
BraA03004341	GCTACATCAAGTAGGCAGACA	AGTATTCACAAGCCCGTTC
BraA03005545	CATTTTCATTTGCTGCGTA	CATTTTCATTTGCTGCGTA
BrOPS	ATGAATCCATCAACGGACCC	TCAATACATCCTCATAGCACTCCT
BrmOPS	ATGAATCCATCAACGGACCC	CTATTTCTGAAGCTTCTTGCTGAA
BrOPS1	ATGAATCCGGCAACTGACCC	TCAATACAGCCTCATAACATTCCTCGC
BrBIN2	ATGCCGGCTGCTGTAGTTG	TTAAGTTCCAGACTGATTCAAGAAACTTAG
BrBES1	ATGAAACATGTTACCGGAGCTCAA	TCAACTACGACCTTTAGAGTTTCCA
BrBES1.3	ATGACGTCTGACGGTGCGA	CTAACGACCTTTGGTGTTTCCAAG
BrBES1.2	ATGACGTCGGACGGCGC	CTACATACGACCTTTAGCGTTTCCA
AtPIP2A	ATGGCAAAGGATGTGGAAGCC	TTAGACGTTGGCAGCACTTCTG
BrAS1pro	AAGCTTAATCCTGTTTTTACCCACC	GGATCCCTCCTGATCCTCCG
BrKNAT4.1pro	AAGCTTTGCCATTTCTTTTAGGGAC	GGATCCCGTTTTCTTTTAAAAAAATCTTC
BrKNAT4.2pro	AAGCTTTGACTCCCTTTGTCCAA	GGATCCTGTTTTCTTTTTTTAACTCGTG

Supplementary Table S2 Primers designed for qRT-PCR

Gene Name	Forward (5’-3’)	Reverse (5’-3’)
BrOPS	AGTTTCTGGTCAGCCGCCTC	TGCCTCCCTATCGGTTTCTCC
BrOPS1	GCAACGTTACCCTCCGATCA	AGCTTCGTTGCTGGAGTTATCA
BrAS1	GAGTTTCGCTGAGAAGCTCGTG	CTCACAGCAACACTGTTCCCG
BrBR6OX2	CTAATCTCTCTAACAAGCCCGGC	ACGGTTTCGAACTCATCCCAAC
BrCPD	GGAAGAAGCCAAAAAGATAACG	GGTAAGTAGTGGAGAAGAGAGGGA
BrCYP90D1	GATATCTCCAAGACGGTTGCA	CCATCTTCTTCTTAGCTTGGAGAG
BrBES1	AAGGTAACTTCAATCTTCCCAGGC	CCAGCCATGTCATCAGGAAGAG
BrDWF4	CGAAGAAGATGAAGCAGAGA	ACAGATGATGTCTCATGTCC
BrACTIN7	AGAGCCGCTTCCTTCAACATCATT	TGGGCACACGGAAGGACATACC

Supplementary Table S3 Primers designed for split-ubiquitin membrane yeast two-hybrid assay

Gene Name	Forward (5’-3’)	Reverse (5’-3’)
BrOPS-sfi	AAGAACGCGGCCATTATGAATCC ATCAACGGACC	CCCCGACATGGCCGAATACATCCTCAT AGCACTCCTCG

BrmOPS-sfi	AAGAACGCGGCCATTATGAATCCATCAACGGACC	CCCCGACATGGCCGATTTCTGAAGCTTCTTGCTGAAAA
BrOPS1-sfi	AAGAACGCGGCCATTATGAATCCGGCAACTGACCC	CCCCGACATGGCCGATCAATACAGCCTCATAACATTCCTCGC
BrBIN2-sfi	CAGAGTGGCCATTACGGCCCGGATGCCGGCTGCTGTAGTTG	CTCGAGAGGCCGAGGCGGCCGTTAAGTTCCAGACTGATTCAAGAAAC

Supplementary Table S4 Primers designed for subcellular localization and BiFC

Gene Name	Forward (5'-3')	Reverse (5'-3')
BrOPS-sl	TGAACTATACAAAGGCGCGCCAATGAATCCATCAACGGACCC	CGCTCTAGAACTAGTTAATTAATCAATACATCCTCATAGCACTCCT
BrmOPS-sl	TGAACTATACAAAGGCGCGCCAATGAATCCATCAACGGACCC	CGCTCTAGAACTAGTTAATTAACTATTTCTGAAGCTTCTTGCTGAA
BrBIN2-sl	CTCTCTCTCAAGCTTGGATCCATGCCGGCTGCTGTAGTTGA	CTCGGAGGAGGCCATGTCGACAGTTCCAGACTGATTCAAGA
AtPIP2A-Rsl	CTCTCTCTCAAGCTTGGATCCATGGCAAAGGATGTGGAAGCC	CTCGGAGGAGGCCATGTCGACGACGTTGGCAGCACTTCTG
AtPIP2A-Gsl	CTCTCAAGCTTGGATCCATGGCAAAGGATGTGGAAGCC	GCCCTTGCTCACCATGTCGACGACGTTGGCAGCACTTCTG
AtWRKY71-sl	CTCTCAAGCTTGGATCCATGGATGATCATGTTGAGCAC	GCCCTTGCTCACCATGTCGACAGACTCGTTCTTGGAGAACAT
BrOPS-B	GCCTATGGAAGTCGACATGAATCCATCAACGGACCC	AGTCACTATGGTCGACTCAATACATCCTCATAGCACTCCT
BrmOPS-B	GCCTATGGAAGTCGACATGAATCCATCAACGGACCC	AGTCACTATGGTCGACCTATTTCTGAAGCTTCTTGCTGAA
BrOPS1-B	GCCTATGGAAGTCGACATGAATCCGGCAACTGACCCAG	AGTCACTATGGTCGACTCAATACAGCCTCATAACATTCCTCGC
BrBIN2-B	TGACTATGCGGTCGACATGCCGGCTGCTGTAGTTG	AGTCACTATGGTCGACTTAAGTTCCAGACTGATTCAAGAAACTTAG

Supplementary Table S5 Primers designed for GUS assay

Gene Name	Forward (5'-3')	Reverse (5'-3')
BrBES1-G	CGGGGGACTCTTGACCATGGATGAAACATGTTACCGGAGCTCAA	AAGTTCTTCTCCTTTACTAGTTCAACTACGACCTTTAGAGTTTCCA
BrBES1.3-G	CGGGGGACTCTTGACCATGGATGACGTCTGACGGTGCGA	AAGTTCTTCTCCTTTACTAGTCTAACGACCTTTGGTGTTTCCAAG
BrBES1.2-G	CGGGGGACTCTTGACCATGGATGACGTCGGACGGCGC	AAGTTCTTCTCCTTTACTAGTCTACATACGACCTTTAGCGTTTCCA

BrAS1pro-G	GACCATGATTACGCCAAGCTTAAGCTTAATCCTGTTTTTACCCACC	GGACTGACCACCCGGGGATCCGGATCCCTCCTGATCCTCCG
BrKNAT4.1 pro-G	GACCATGATTACGCCAAGCTTCCCAAGCTTAAGCTTTGCCATTTCTTTTAGGGAC	GGACTGACCACCCGGGGATCCGGATCCCGTTTTCTTTTAAAAAAATCTTC
BrKNAT4.2 pro-G	GACCATGATTACGCCAAGCTTCCCAAGCTTAAGCTTTGACTCCCTTTGTCCAA	GGACTGACCACCCGGGGATCCGGATCCTGTTTTCTTTTTTTAACTCGTG

Supplementary Table S6 BrOPS interacting proteins identified by split-ubiquitin membrane yeast two-hybrid assay screening

Putative protein type	Frequency	Gene ID
GSK3-like kinase BIN2	6	BraA01001098
NADPH-dependent thioredoxin reductase 1	2	BraA03006362
40S RIBOSOMAL PROTEIN SA	2	BraA07004210
Cytochrome P450	2	BraA10000836
Nucleoside diphosphate kinase family protein	2	BraA03005492
Kinase interacting (KIP1-like) family protein	1	BraA09000118
Calcium-dependent lipid-binding (CaLB domain) family protein	1	BraA09000331
Endomembrane protein 70 protein family	1	BraA06002292
Phosphate transporter 1;5,	1	BraA03001848

Supplementary Table S7 Probes of *BrAS1* promotor for EMSA

Probe	Sequence
WT	CGATCTTTAAAGTTTTGATCTTTTTGGACGCAGATGAAGAAGGAATGGTGTTGAGATGGGAAGGAG
MT	CGATCTTTAAAGTTTTGATCTTTTTGGACGTGAGCAAAGAAGGAATGGTGTTGAGATGGGAAGGAG

Supplementary Table S8 Primers designed for EMSA and pull-down assay

Gene Name	Forward (5'-3')	Reverse (5'-3')
BrBES1-E	CATCACGGGAGCGGCGGATCCATGAAACATGTTACCGGAGCTCAA	TCGCCCTTGCTCACCAAGCTTTCAACTACGACCTTTAGAGTTTCCA
BrOPS-P	CATCACGGGAGCGGCGGATCCATGAATCCATCAACGGACCC	TCGCCCTTGCTCACCAAGCTTTCAATACATCCTCATAGCACTCCT
BrBIN2-P	CATCACGGGAGCGGCGGATCCATGCCGGCTGCTGTAGTTG	TCGCCCTTGCTCACCAAGCTTTTAAGTTCCAGACTGATTCAAGAAACTTAG

Supplementary Table S9 Primers designed for Y1H system

Gene Name	Forward (5’-3’)	Reverse (5’-3’)
BrBES1-AD	CCAGATTACGCTCATATGAAACATGTTACCGGAGCTCAA	CCCACCCGGGTGGAATTCTCAACTACGACCTTTAGAGTTTCCA
BrBES1.2-AD	CCAGATTACGCTCATATGACGTCGGACGGCGC	CCCACCCGGGTGGAATTCCTACATACGACCTTTAGCGTTTCCA
BrBES1.3-AD	CCAGATTACGCTCATATGACGTCTGACGGTGCGA	CCCACCCGGGTGGAATTCCTAACGACCTTTGGTGTTTCCAAG
BrAS1pro-Y	AAGCTTGAATTCGAGCTCAAGCTTAATCCTGTTTTTACCCACC	CATGCCTCGAGGTCGACGGATCCCTCCTGATCCTCCG
BrKNAT4.1pro-Y	AAGCTTGAATTCGAGCTCAAGCTTTGCCATTTCTTTTAGGGAC	CATGCCTCGAGGTCGACGGATCCCGTTTTCTTTTAAAAAAATCTTC
BrKNAT4.2pro-Y	AAGCTTGAATTCGAGCTCAAGCTTTGACTCCCTTTGTCCAA	CATGCCTCGAGGTCGACGGATCCTGTTTTCTTTTTTTAACTCGTG

Supplementary Table S10 Primers designed for LUC system

Gene Name	Forward (5’-3’)	Reverse (5’-3’)
BrBIN2-nL	ACGGGGGACGAGCTCGGTACCATGCCGGCTGCTGTAGTTG	CGCGTACGAGATCTGGTCGACAGTTCCAGACTGATTCAAGAAACTTAG
BrOPS-cL	TACGCGTCCCGGGGCGGTACCATGAATCCATCAACGGACCC	ACGAAAGCTCTGCAGGTCGACTCAATACATCCTCATAGCACTCCT
BrAS1pro-L	CTATAGGGCGAATTGGGTACCAAGCTTAATCCTGTTTTTACCCACC	TCCAGTCCGCGGTGAGCGGCCGCGGATCCCTCCTGATCCTCCG

Supplementary Table S11 Primers for transgenic insert and genotype identification

Gene Name	Forward (5’-3’)	Reverse (5’-3’)
35S-OPS	GACGCACAATCCCACTATCC	CGCTGCTCGTCTTGGCTAAAC
bri1-116	CAATCTTAACTGGATTTCTCTGTC	CATCGGAACCATTGTTATCAAACGTC

References for SI reference citations

1. Y. Lu *et al.*, Microspore induced doubled haploids production from ethyl methanesulfonate (EMS) soaked flower buds is an efficient strategy for mutagenesis in Chinese cabbage. *Front. Plant. Sci.* 7, 1780 (2016).
2. H. Li, R. Durbin, Fast and accurate short read alignment with Burrows-Wheeler transform. *Bioinformatics.* 25, 1754-1760 (2009).
3. A. Abe *et al.*, Genome sequencing reveals agronomically important loci in rice using MutMap. *Nat. Biotechnol.* 30, 174-178 (2012).
4. K. Semagn, R. Babu, S. Hearne, M. Olsen, Single nucleotide polymorphism genotyping using Kompetitive Allele Specific PCR (KASP): overview of the technology and its application in crop improvement. *Mol. Breeding.* 33, 1-14 (2014).
5. J. Yang *et al.*, Target SSR-seq: a novel SSR genotyping technology associate with perfect SSRs in genetic analysis of cucumber varieties. *Front. Plant. Sci.* 10, 531 (2019).
6. Y. Onda, K. Takahagi, M. Shimizu, K. Inoue, K. Mochida, Multiplex PCR targeted amplicon sequencing (MTA-Seq): simple, flexible, and versatile SNP genotyping by highly multiplexed PCR amplicon sequencing. *Front. Plant. Sci.* 9, 201 (2018).
7. W. Zhao *et al.*, RNA-Seq-based transcriptome profiling of early nitrogen deficiency response in cucumber seedlings provides new insight into the putative nitrogen regulatory network. *Plant. Cell. Physio.l* 56, 455-467 (2014).
8. A. Gu *et al.*, Coupling Seq-BSA and RNA-Seq analyses reveal the molecular pathway and genes associated with heading type in Chinese cabbage. *Front. Genet.* 8, 176 (2017).
9. Y. J. Yoo *et al.*, Interactions between transmembrane helices within monomers of the aquaporin AtPIP2;1 play a crucial role in tetramer formation. *Mol. Plant.* 9, 1004-1017 (2016).

10. D. Guo *et al.*, The WRKY Transcription Factor WRKY71/EXB1 Controls Shoot Branching by Transcriptionally Regulating *RAX* Genes in Arabidopsis. *Plant. Cell.* 27, 3112-27 (2015).
11. L. Du, *et al.*, The ubiquitin receptor DA1 regulates seed and organ size by modulating the stability of the ubiquitin-specific protease UBP15/SOD2 in *Arabidopsis*. *Plant. Cell*. 26, 665-677 (2014).
12. E. Scarpella, P. Francis, T. Berleth, Stage-specific markers define early steps of procambium development in Arabidopsis leaves and correlate termination of vein formation with mesophyll differentiation. *Development,* 131, 3445-3455 (2004).
13. M. Blázquez., Quantitative GUS Activity Assay in Intact Plant Tissue. *CSH. Protoc*. 2, pdb-prot4688 (2007).
14. D. Kusumoto *et al.*, Resistance of Red Clover (*Trifolium pratense*) to the Root Parasitic Plant *Orobanche minor* is Activated by Salicylate but not by Jasmonate. *Ann. Bot.* 100, 537–544 (2007).
15. D. A. Johansen, Plant microtechnique. New York: McGraw-Hill (1940).
16. W. Li *et al.*, Sclerenchyma cell thickening through enhanced lignification induced by OsMYB30 prevents fungal penetration of rice leaves. *New. Phytol.* 226, 1850-1863 (2020).
17. L. Zhang, W. Q. Liu, J. Li, Establishing a Eukaryotic Pichia pastoris Cell-Free Protein Synthesis System. *Front. Bioeng. Biotechnol.* 8, 536 (2020).
18. Z. Qin *et al.*, Divergent roles of FT-like 9 in flowering transition under different day lengths in *Brachypodium distachyon*. *Nat. Commun.* 10, 812 (2019).
19. R. P. Hellens *et al.*, Transient expression vectors for functional genomics, quantification of promoter activity and RNA silencing in plants. *Plant. Methods.* 1, 13 (2005).
20. S. Solanki *et al.*, Visualization of spatial gene expression in plants by modified RNAscope fluorescent in situ hybridization. *Plant. Methods.* 16, 71 (2020).
21. M. D. Abràmoff, P. J. Magalhães, S. J. Ram, Image Processing with ImageJ. *Biophotonics*. Int. 11, 36-42 (2004).

22. K. Caesar *et al.*, A fast brassinolide-regulated response pathway in the plasma membrane of *Arabidopsis thaliana*. *Plant. J.* 66, 528-540 (2011).
23. S. D. Yoo, Y. H. Cho, J. Sheen, Arabidopsis mesophyll protoplasts: a versatile cell system for transient gene expression analysis. *Nat. Protoc.* 2, 1565-1572 (2007).
24. S. J. Clough, A. F. Bent, Floral dip: a simplified method for Agrobacterium-mediated transformation of *Arabidopsis thaliana*. *Plant. J.* 16, 735-743 (1998).
